# Antihypertension Nanoblockers Increase Intratumoral Perfusion of Sequential Cytotoxic Nanoparticles to Enhance Chemotherapy Efficacy against Pancreatic Cancer

**DOI:** 10.1002/advs.202201931

**Published:** 2022-08-26

**Authors:** Suchen Bian, Haijiang Dong, Long Zhao, Zequn Li, Jian Chen, Xingxin Zhu, Nasha Qiu, Xing Jia, Wenfeng Song, Zekuan Li, Shusen Zheng, Hangxiang Wang, Penghong Song

**Affiliations:** ^1^ Division of Hepatobiliary and Pancreatic Surgery Department of Surgery The First Affiliated Hospital Zhejiang University School of Medicine Hangzhou 310003 China; ^2^ NHC Key Laboratory of Combined Multi‐organ Transplantation Hangzhou 310003 China; ^3^ Key Laboratory of the diagnosis and treatment of Organ Transplantation Research Unit of Collaborative Diagnosis and Treatment for Hepatobiliary and Pancreatic Cancer Chinese Academy of Medical Sciences (2019RU019) Hangzhou 310003 China; ^4^ Key Laboratory of Organ Transplantation Research Center for Diagnosis and Treatment of Hepatobiliary Diseases Hangzhou Zhejiang Province 310003 China; ^5^ Department of Hepatobiliary and Pancreatic Surgery The Center for Integrated Oncology and Precision Medicine Affiliated Hangzhou First People's Hospital Zhejiang University School of Medicine Hangzhou 310006 China

**Keywords:** losartan, pancreatic ductal adenocarcinoma, prodrug self‐assembly, tumor microenvironment, tumor penetration

## Abstract

Pancreatic ductal adenocarcinoma (PDAC), one of the worst prognosis types of tumors, is characterized by dense extracellular matrix, which compresses tumor vessels and forms a physical barrier to inhibit therapeutic drug penetration and efficacy. Herein, losartan, an antihypertension agent, is applied as a tumor stroma modulator and developed into a nanosystem. A series of lipophilic losartan prodrugs are constructed by esterification of the hydroxyl group on losartan to fatty acids. Based on the self‐assembly ability and hydrodynamic diameter, the losartan‐linoleic acid conjugate is selected for further investigation. To improve the stability in vivo, nanoassemblies are refined with PEGylation to form losartan nanoblocker (Los NB), and administered via intravenous injection for experiments. On murine models of pancreatic cancer, Los NB shows a greater ability to remodel the tumor microenvironment than free losartan, including stromal depletion, vessel perfusion increase, and hypoxia relief. Furthermore, Los NB pretreatment remarkably enhances the accumulation and penetration of 7‐ethyl‐10‐hydroxycamptothecin (SN38)‐loaded nanodrugs (SN38 NPs) in tumor tissues. Expectedly, overall therapeutic efficacy of SN38 NPs is significantly enhanced after Los NB pretreatment. Since losartan is one of the most commonly used antihypertension agents, this study may provide a potential for clinical transformation in stroma‐rich PDAC treatment.

## Graphical abstract

Self‐assembled antihypertension losartan (Los)‐fatty acid prodrugs are constructed. Los‐linoleic acid conjugate is chosen and PEGylated to afford Los nanoblocker (NB). In the pancreatic cancer mouse model, intravenously injected Los NB remodels stromal tumor microenvironment, improves the intratumoral accumulation and penetration of sequentially administered cytotoxic 7‐ethyl‐10‐hydroxycamptothecin (SN38) nanotherapy, and results in the superior antitumor activity.

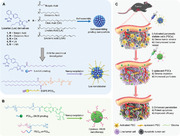

## 1 Introduction

Pancreatic ductal adenocarcinoma (PDAC) is a lethal disease with overall survival typically less than 6 months from diagnosis.^[^ ^1^ ^]^ PDAC shows no obvious symptoms until reaching an advanced stage, which makes its early diagnosis very difficult.^[^ ^2^ ^]^ Approximately 80% of PDAC patients are diagnosed at an advanced stage or found to have distant metastases at the initial diagnosis; thus, only a few patients are eligible for surgery.^[^ ^3^ ^]^ Chemotherapy remains a mainstay treatment to reduce the mortality of PDAC patients. Unfortunately, PDAC is characterized as one of the most stroma‐rich cancers, consisting of more than 90% stroma in tumors.^[^ ^4^ ^]^ Tumor vessels compressed by the dense fibrotic stroma contribute to heterogenous perfusion and limited therapeutic delivery in PDAC. More seriously, high interstitial solid stress and abundant stroma form the second physical and biological barrier, further restricting the entrance of therapeutics.^[^ ^5^ ^]^ A large cohort of PDAC patients do not respond to chemotherapies but still experience substantial side effects. Therefore, PDAC is in urgent need of effective new therapies in clinic.

Cancer nanomedicines hold great potential to overcome the limitations of free chemotherapeutic drugs, thereby improving the balance between their efficacy and toxicity.^[^ ^6^ ^]^ Nevertheless, the tumor tissue accumulation and therapeutic effect of nanomedicines for PDAC treatment remain unsatisfactory, resulting from the dense desmoplastic stroma of pancreatic cancer.^[^ ^7^ ^]^ Thus, stroma‐targeting therapy in PDAC based on nanodrug delivery systems have received much attention. For example, vasodilator hydralazine liposomes were reported to reduce tumor stroma and enlarge tumor vessels for enhancing accumulation of liposomal doxorubicin in melanoma.^[^ ^8^ ^]^ Quercetin‐based approach to remodel the stroma‐rich tumor microenvironment (TME) using nanoparticles showed an effect on improving penetration and antitumor effect of cisplatin nanoparticles in bladder cancer.^[^ ^9^ ^]^ Losartan, a clinically widely approved drug for antihypertension, has been recently reported to have a remarkable effect on reducing stroma levels in tumors.^[^ ^10^ ^]^ Losartan can perform as the blocker of the transforming growth factor *β* (TGF‐*β*) pathway, which performs an essential function in the secretion of excessive extracellular matrix (ECM) proteins.^[^ ^11^ ^]^ A losartan pretreatment strategy has been reported to improve the tumor photodynamic treatment efficacy of nanoplatforms.^[^ ^12^ ^]^ Therefore, losartan‐based nanodelivery system could be an attractive TME modulating strategy for enhancing the accumulation and therapeutic efficacy of chemotherapeutic nanomedicines in PDAC treatment, which has never been reported.

In this study, we proposed losartan‐based nanoparticles via small‐molecule assembling strategy. Specifically, a series of lipophilic losartan prodrugs were constructed by esterification of the hydroxyl group on losartan to fatty acids, possessing the advantages of high drug loading capacity, facile synthesis process, and efficient fabrication processes.^[^ ^13^ ^]^ Based on the self‐assembly ability and hydrodynamic diameter (*D*_H_), the losartan‐linoleic acid (Los‐LA) conjugate was selected for further investigation. To improve the stability in vivo, nanoassemblies were refined with PEGylation to form Los‐LA nanoparticles, that we named Los nanoblocker (NB), and administered via intravenous injection. To mimic the pathohistological features and in situ progression of human PDAC, we established a patient‐derived xenograft (PDX) subcutaneous pancreatic cancer mouse model and a Panc02 orthotopic pancreatic cancer mouse model. The effect of Los NB on TME modulation was evaluated, including the stroma level, vessel perfusion, and tumor hypoxia. Then, 7‐ethyl‐10‐hydroxycamptothecin (SN38)‐loaded nanodrugs were applied as sequential therapeutic nanodrugs, which have been previously published by our group,^[^ ^14^ ^]^ and whether their delivery will be increased by prior administration of Los NB was studied. Through remodeling the TME, Los NB were expected to improve the penetration and therapeutic efficacy of SN38‐loaded nanodrugs in pancreatic tumors.

## 2 Results and Discussion

### 2.1 Design of the Prodrugs and their Self‐Assembly Behavior in Water

**Figure 1:**
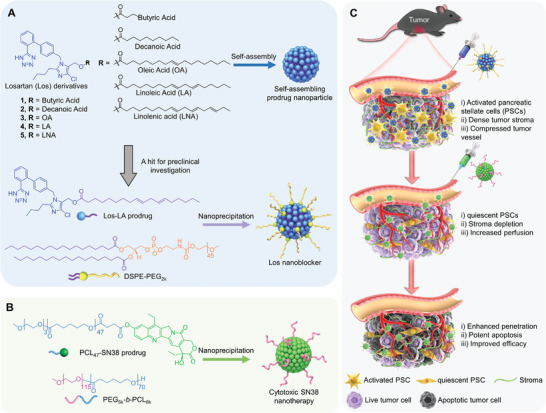
Self‐assembly of antihypertension losartan (Los) prodrugs to increase the nanodelivery and efficacy of cytotoxic camptothecin nanotherapy in pancreatic cancer. A) Chemical structures of Los prodrugs 1–5 and self‐assembly of the prodrugs. Prodrugs were constructed by esterification of the hydroxyl group on Los to fatty acids. Optimization of the prodrugs in terms of self‐assembling behavior allows to identify a hit (i.e., Los‐LA conjugate) for in vivo investigation. The self‐assembled Los‐LA conjugate was PEGylated using DSPE‐PEG_2k_ to afford Los nanoblocker (NB). B) Chemical structures of polymeric 7‐ethyl‐10‐hydroxycamptothecin (SN38) prodrug and the polymer matrix for generation of cytotoxic SN38 nanotherapy. C) Schematic illustration of a sequential regime to treat PDAC. Intravenously injected Los NB remodels TME through converting activated pancreatic stellate cells into quiescence, depleting the dense tumor stromal barrier, and increasing tumor vessel perfusion. Eventually, this improves the intratumoral accumulation and penetration of sequentially administered cytotoxic SN38 nanotherapy and results in the superior antitumor activity.

**Figure 2:**
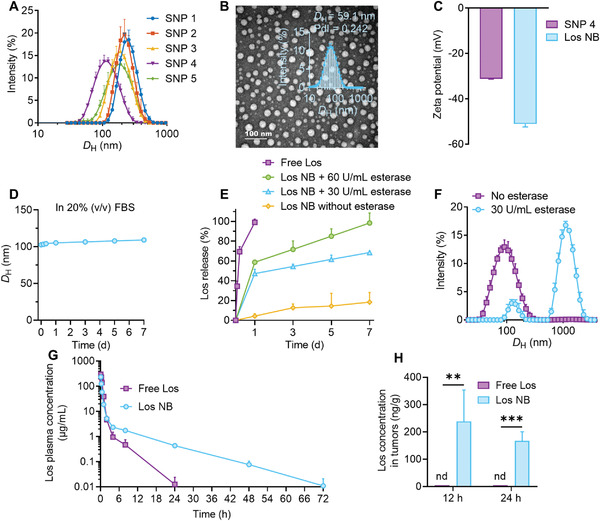
Characterization of losartan prodrug‐assembled nanoblockers. A) Diameter distributions of self‐assembled losartan prodrugs (*n* = 3). B) Representative TEM image and size distribution of Los NB (*n* = 3). C) Zeta potentials of self‐assembled Los‐LA prodrug (SNP 4) and Los NB (*n* = 3). D) Stability of Los NB in 20% v/v FBS, determined by changes in size (*n* = 3). E) In vitro losartan release profiles from free losartan and Los NB against PBS, and Los NB against PBS containing esterase (30 or 60 U mL^−1^) (*n* = 3). F) Change in the size distribution of Los NB after incubation with 30 U mL^−1^ esterase (*n* = 3). G) Plasma drug concentration–time profiles of free losartan and Los NB after a single intravenous injection in SD rats (*n* = 3). H) Drug concentration in tumors at 12 and 24 h postadministration (*n* = 5). Data are presented as mean ± SD. Significance was assessed by student's *T*‐test. ns, *p* > 0.05, *p < 0.05, **p < 0.01, ***p < 0.001.

We and other groups previously demonstrated that covalent conjugation of active compounds to lipophilic fatty acids could endow the prodrug entities with amphiphilicity and enabled them to self‐assemble into nanoparticles in aqueous media without exogenous excipients.^[^ ^15^ ^]^ Inspired by these findings, we here constructed a small panel of lipophilic losartan prodrugs by esterification of the hydroxyl group on losartan to fatty acids such as butyric acid, decanoic acid, oleic acid (OA), linoleic acid (LA), and linolenic acid (LNA) (**Figure** **1**A and Schemes S1–S5, Supporting Information). The syntheses of these compounds proceeded in moderate to good yields without tedious procedures. To evaluate the self‐assembly ability, prodrug solutions in dimethyl sulfoxide (DMSO) were injected into deionized (DI) water under ultrasonication. This protocol allowed us to identify that the prodrugs **3**, **4**, and **5** tethered with OA, LA, and LNA, respectively, were capable of forming stable self‐assembling nanostructures rather than precipitates (Figure S3, Supporting Information). By sharp contrast, the prodrugs **1** and **2** with short alkyl chains were amenable to precipitate in water. This is possibly attributed to structural flexibility and intermolecular *π*–*π* stacking of polyunsaturated alkyl chains, which resulted in entropy reduction.^[^ ^16^ ^]^ Comparatively, nanoassemblies prepared from compound **4** had a minimum hydrodynamic diameter and higher stability compared with other nanoassemblies, as determined by dynamic light scattering (DLS) analysis (**Figure** **2**A). We therefore chose prodrug **4** (Los‐LA) for surface polyethylene glycol (PEG) cloaking and subsequent in vivo experiments.

### 2.2 Characterization of the PEGylated Los‐LA Nanoparticles

To reduce macrophage uptake and prolong circulation when systemically injected, 1,2‐distearoyl‐*sn*‐glycero‐3‐phosphoethanolamine‐*N*‐[methoxy (polyethylene glycol) 2000] (DSPE‐PEG_2k_) was used for surface PEG cloaking of nanoparticles (Figure 1A).^[^ ^17^ ^]^ To prepare PEGylated Los‐LA nanoblockers (referred to as Los NB), a solution of Los‐LA and DSPE‐PEG_2k_ at 10:1 w/w dissolved in DMSO was injected into DI water following the same protocol. Compared with the self‐assembled prodrug, PEGylated Los NB had a smaller diameter, as measured by transmission electron microscopy (TEM) (30.9 ± 3.7 nm vs 95.6 ± 19.9 nm) and DLS analysis (59.1 nm ± 1.6 vs 128.3 ± 1.3 nm) (Figure 2B). The small size (<100 nm) may be beneficial for effective penetration of Los NB into hypovascular and hypopermeable PDAC tissues.^[^ ^18^ ^]^ Compared with uncoated SNP **4**, PEGylated Los NB presented a more negative surface charge, suggesting higher stability resulting from electrostatic repulsion (Figure 2C). The *D*_H_ of Los NB in water increased from 59.1 ± 1.6 to 89.0 ± 2.5 nm within 1 day (Figure S4A, Supporting Information), which might be due to the formation of the hydration layer, since TEM imaging showed no significant change in morphology and particle size of Los NB with the prolonged preservation time (Figure S4B,C, Supporting Information).^[^ ^19^ ^]^ Moreover, no significant change in *D*_H_ was observed from Day 2 to Day 14 (Figure S4D, Supporting Information). When incubated with 20% fetal bovine serum (FBS), the *D*_H_ distribution of Los NB was not significantly altered within 6 days, indicating its satisfactory dimensional stability (Figure 2D and Figure S5, Supporting Information). The release kinetics studies revealed that Los NB has a sustained release rate of losartan, with only ≈18.4% release after 1 week of dialysis (Figure 2E). Comparatively, free losartan physically encapsulated in DSPE‐PEG_2k_ micelles has rapidly released nearly 100% of losartan after 1 day (Figure 2E). The good stability and sustained‐release characteristic of Los NB may inhibit the burst release of therapeutics in the blood circulation and increase delivery to tumors. Notably, benefiting from the small‐molecule assembling strategy and marginal use of matrices, Los NB achieved a high encapsulation efficiency (EE) of 92.5 ± 0.6% and a high drug loading (DL) of 51.9 ± 0.3%.

In addition, the ester bond used for drug conjugation is susceptible to intracellular esterase, ensuring that the active drug can be liberated properly. As expected, in the presence of porcine liver esterase (PLE), the release of active losartan from Los NB was much more rapid (≈47.3% release with 30 U mL^−1^ PLE and ≈58.8% release with 60 U mL^−1^ PLE within 1 day) (Figure 2E). Moreover, the ratio of released losartan to Los‐LA from Los NB was analyzed. Upon exposure to PLE, the drugs released were dominated by losartan, which further confirmed that the ester bond of Los‐LA prodrug was hydrolyzed efficiently (Figure S6, Supporting Information). In addition to these release observations, variation in size distribution and formation of large aggregates after incubation with PLE was also confirmed by DLS analysis, which could be due to esterase‐responsive drug release and structural disruption (Figure 2F). LA is an essential fatty acid for human health, losartan is extensively used in the clinic with low toxicity, and DSPE‐PEG has been approved by Food and Drug Administration for human use; we thus envision that Los NB possesses favorable biocompatibility and high translation capacity to the clinic.

A pharmacokinetic study was performed to verify the superiority of Los NB in prolonging blood circulation. A single dose of free losartan or Los NB (20 mg kg^−1^ losartan equivalent dose) was intravenously injected into Sprague–Dawley (SD) rats. Blood samples collected at predetermined time points were subjected to liquid chromatography mass spectrometry (LCMS) analysis. As the plasma drug concentration–time curves shown in Figure 2G, free losartan was rapidly cleared within 24 h (*t*_1/2_: 1.85 ± 0.78 h), whereas Los NB prolonged losartan circulation time to 72 h (*t*_1/2_: 7.03 ± 0.64 h). In addition, we analyzed the tumor tissue distribution of Los NB on the Panc02 orthotopic pancreatic cancer mouse model. A single dose of free losartan or Los NB (20 mg kg^−1^ losartan equivalent dose) was intravenously injected. Tumor tissues were taken at 12 or 24 h after injection, and losartan concentration was detected by LCMS. Losartan was unable to be detected in tumor tissue at 12 h in the free losartan group, while Los NB showed persistent tumor accumulation (Figure 2H). Furthermore, Los NB was labeled with 1,1’‐dioctadecyl‐3,3,3’,3’‐tetramethylindotricarbocyanine iodide (DiR), a lipophilic near‐infrared fluorescence probe. Tumor tissues were harvested 24 h after injection. Fluorescence observation of tumor frozen sections indicated the greater penetration of DiR‐labeled Los NB than free DiR (Figure S7, Supporting Information). In conclusion, Los NB showed superiorities in prolonging blood circulation and increasing tumor accumulation and was expected to have stronger tumor stroma modulation efficacy than free losartan.

### 2.3 Los Nanoblocker Reversed the Stroma‐Rich PDAC TME

First, tumor stroma modulation efficacy of Los NB was validated in vitro using murine pancreatic adenocarcinoma cells Panc02 and murine embryonic fibroblasts NIH‐3T3. To acquire similar characteristics and functions to tumor‐associated fibroblasts, NIH‐3T3 fibroblasts were preactivated by incubating with TGF‐*β*1 for 96 h at a dose of 10 ng mL^−1^.^[^ ^20^ ^]^ Both free losartan and Los‐LA showed no significant cytotoxicity against Panc02 or NIH‐3T3 cells at the highest concentration of 50 × 10^−6^ m (losartan equivalent) within 24 h, indicating that the tumor stroma modulation efficacy of Los NB does not depend on cytotoxic capacity (Figure S8A,B, Supporting Information). Then, the esterase activity of Panc02 and NIH‐3T3 cells was measured with fluorescein diacetate, a cell‐permeating esterase substrate. As shown in Figure S8C,D, Supporting Information, both Panc02 and NIH‐3T3 cells have high esterase activity, and the esterase activity of NIH‐3T3 was only slightly lower than Panc02, suggesting that active losartan can be liberated properly from Los NB in Panc02 and NIH‐3T3 cells. It has been reported that both pancreatic cancer cells and tumor‐associated fibroblasts can release TGF‐*β* that activates pancreatic stellate cells (tumor‐associated fibroblasts) to secrete ECM proteins.^[^ ^21^ ^]^ To compare the stroma modulation activity between free losartan, Los‐LA, and Los NB, Panc02 and NIH‐3T3 cells were incubated with the same concentration of free losartan/Los‐LA/Los NB (20 × 10^−6^ m losartan equivalent) for 24 h. Consistent with existing studies, free losartan significantly inhibited the TGF‐*β* secretion of Panc02 and NIH‐3T3 cells (Figure S8E–G, Supporting Information), suppressed the expression of *α*‐smooth muscle actin (*α*‐SMA), a biomarker of activated pancreatic stellate cells (Figure S8H, Supporting Information), and fibronectin of NIH‐3T3 cells (Figure S8I, Supporting Information).^[^ ^22^ ^]^ Noteworthily, a similar level of stroma modulation efficacy was observed in Los‐LA and Los NB, suggesting our hypothesis that the rapid esterase responsiveness of Los NB contributes to its modulation efficacy on cancer cells and tumor‐associated fibroblasts.

**Figure 3:**
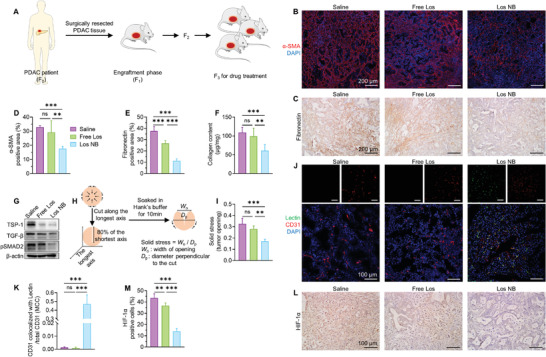
TME changes in PDX tumors after saline/free Los/Los NB treatments. A) Illustration of the establishment of the PDX mouse model. B–E) Representative images and quantitative analysis of immunohistochemistry staining of *α*‐SMA and fibronectin (*n* = 5). F) Collagen content in tumor tissues (*n* = 5). G) Western blot results of the expression of TSP‐1, TGF‐*β*, and pSMAD2 in tumor tissues. H) Schematic representation of solid stress measurement. I) Solid stress of tumor tissues (*n* = 5). J,K) Tumor vessel perfusion indicated by the percentage of perfused vessels (anti‐CD31 staining colocalized with lectin) among total vessels (total CD31) (*n* = 5). L,M) Representative images and quantitative analysis of hypoxia (anti‐HIF‐1*α* staining) in tumor tissues (*n* = 5). Five randomly chosen visual fields were evaluated for histological quantification. Data are presented as mean ± SD. Significance was assessed by one‐way ANOVA followed by LSD post hoc test. ns, *p* > 0.05, *p < 0.05, **p < 0.01, ***p < 0.001.

Although cancer cell‐derived xenograft (CDX) models have been widely used for preclinical drug evaluation, the deficiency in TME presentation and difference from tumors in the clinic might lead to unreliable results, especially in pancreatic cancer characterized by a dense ECM and heterogenous TME.^[^ ^23^ ^]^ Therefore, to mimic the pathohistological and genetic features of original human tumor tissues, we first used a rodent model of PDAC patient‐derived xenograft. Fresh human PDAC tumor tissue was subcutaneously implanted into immune‐deficient nude mice, and the PDX tumor was passaged to the next nude mice. The third generation (F_3_) of mice was used for our experiments, which had been verified to have highly consistent tumor characteristics with the primary patient generation (F_0_) (**Figure** **3**A).^[^ ^24^ ^]^ PDX tumor tissues showed high expression of *α*‐SMA, a biomarker of activated pancreatic stellate cells (PSCs), and fibronectin and were considered to retain the dense stromal features of clinical PDAC tumor tissues.

Following the establishment of the PDX mouse model, we then tested the efficacy of Los NB. Treatment of Los NB (20 mg kg^−1^ losartan equivalent dose, once daily for 7 days, intravenous injection) significantly downregulated tumor *α*‐SMA and fibronectin expression, while an equal dose of losartan potassium (free Los) treatment showed a limited effect (Figure 3B–E). The effect of Los NB on tumor collagen content reduction was more remarkable than that of free Los (Figure 3F). To further validate the molecular mechanism, we detected the protein expression levels of TGF‐*β*, the key mediator of PSCs activation and ECM protein secretion,^[^ ^25^ ^]^ its upstream effector TSP‐1 and its downstream effector phospho‐Smad2 (pSmad2). Consistent with prior studies,^[^ ^11^, ^26^ ^]^ free Los showed a downregulation effect on the TGF‐*β* pathway, while Los NB displayed more remarkable effects (Figure 3G). Increased stiffness of the ECM has been shown to promote tumor malignancy, chemotherapy tolerance, and invasion.^[^ ^27^ ^]^ Therefore, antistroma treatment may have a role of reducing tumor progression.^[^ ^28^ ^]^ Excessive ECM protein forms a dense cross‐linked mesh and increases solid stress of tumors, which plays a significant role in tumor invasion and metastasis.^[^ ^29^ ^]^ We here assessed whether Los therapies had an impact on the stroma reduction by measuring solid stress of tumors according to the published protocol (Figure 3H).^[^ ^30^ ^]^ Interestingly, the Los NB treatment significantly reduced solid stress in tumors compared with free Los (Figure 3I). These observations supported the excellent tumor stroma reduction of using Los NB.

Excessive ECM deposition causes tumor vascular compression and leads to hypovascularity and hypoxia of the tumor. Thus, we measured the tumor vessel perfusion and hypoxia level after various treatments. Tumors in the untreated group and free Los group were severely hypoperfused, while the Los NB treatment significantly improved the perfused vessel fraction to 47.0 ± 10.6% (Figure 3J,K). The increased tumor vascular perfusion could provide favorable conditions for subsequent drug penetration across tumors.^[^ ^31^ ^]^ In addition, tumors treated with Los NB showed low expression of hypoxia‐inducible factor‐1*α* (HIF‐1*α*), an upregulated factor under hypoxic conditions, suggesting that tumor hypoxia was successfully alleviated through improving vascular perfusion (Figure 3L). Since tumor hypoxia is known as one of the repressor factors for chemotherapy, the Los NB pretreatment might also enhance the efficacy of subsequent chemotherapy.^[^ ^32^ ^]^

Moreover, to mimic the in situ progression of human PDAC, we constructed an orthotopic pancreatic cancer model by injecting mouse Panc02 pancreatic cancer cells into the pancreatic tail of C57BL/6 mice.^[^ ^33^ ^]^ Panc02 tumor is an aggressive malignancy widely used for preclinical studies.^[^ ^34^ ^]^ Consistent with the results in the PDX model, Los NB outperformed free Los in terms of impairing tumor stroma synthesis and remodeling TME (Figure S9, Supporting Information). In addition, improving vascular perfusion has been reported to facilitate intratumoral infiltration of antitumor immune cells.^[^ ^35^ ^]^ In this orthotopic model, we indeed observed that the number of tumor‐infiltrated CD3^+^ T cells, CD8^+^ T cells, and F4/80^+^ macrophages were significantly enhanced by Los NB (Figure S10A–F, Supporting Information). Besides, the elevated levels of several antitumor immune factors including IL‐2, IL‐12p70, and TNF‐*α* were detected in Panc02 tumor tissues after the Los NB treatment (Figure S10G–I, Supporting Information).^[^ ^36^ ^]^ This finding may motivate further studies to explore antihypertension blockers for enhancing the immunotherapy efficiency against cancer. Together, these results demonstrated the potential of a self‐assembled antihypertension nanoblocker for tumor stroma reduction and TME modulation.

### 2.4 Pretreatment of Antihypertension Nanoblocker Enhanced Subsequent Nanotherapy Accumulation

**Figure 4:**
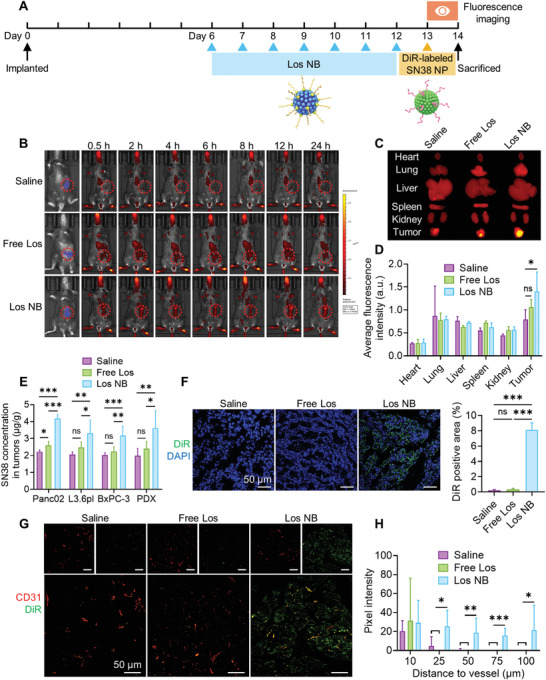
In vivo biodistribution of DiR‐labeled SN38 NPs on orthotopic Panc02 cancer CDX model after Los NB pretreatment. A) Treatment and observation schedule. B) Representative fluorescence images of DiR‐labeled SN38 NPs at predetermined time points. The red signals indicate DiR. The tumor sites are indicated as blue signals in the first column and marked by the red circles. C,D) Representative ex vivo fluorescence images and quantitative analysis of DiR‐labeled SN38 NP distribution in the heart, lungs, liver, spleen, kidney, and tumor harvested at 24 h postadministration (*n* = 3). E) SN38 concentration in tumor tissues on orthotopic Panc02, subcutaneous L3.6pl and BxPC‐3 cancer CDX models plus the PDX model at 24 h postadministration detected by HPLC (*n* = 5). F) Penetration of DiR‐labeled SN38 NPs in tumor tissues at 24 h postadministration (*n* = 5). G) Histological analysis of DiR‐labeled SN38 NPs (green) and tumor vessels (red). H) Average pixel intensity of DiR at the assigned distance from tumor vessels (*n* = 5). Five randomly chosen visual fields were evaluated for histological quantification. Data are presented as mean ± SD. Significance was assessed by one‐way ANOVA followed by LSD post hoc test. ns, *p* > 0.05, *p < 0.05, **p < 0.01, ***p < 0.001.

Inspired by the effects of the nanoblocker on modulation of tumor stroma and enhanced vascular perfusion, we hypothesized that the Los NB pretreatment may facilitate subsequent nanotherapy accumulation and penetration deep into tumor tissues. To test our assumption, an esterase‐activatable camptothecin nanoparticle (termed SN38 NP) was employed as the cytotoxic nanotherapy.^[^ ^14^ ^]^ For in vivo tracing, we labeled SN38 NP with DiR. Panc02 cells expressing firefly luciferase (Panc02‐Luc) were inoculated into the pancreatic tail of C57BL/6 mice and allowed to grow for observation of intratumoral delivery of SN38 NP. Seven injections of Los NB were given to the mice, and then DiR‐loaded SN38 NP was injected to track in vivo biodistribution using fluorescence imaging (**Figure** **4**A). Tumor accumulation of DiR‐loaded SN38 NPs showed a limited enhancement over 24 h in the saline and free Los groups. In contrast, the DiR signal was much stronger at the tumor site and was still maintained at a high level until 24 h in the Los‐LA group (Figure 4B). 24 h after administration, ex vivo images suggested that the Los NB pretreatment significantly increased the accumulation of DiR‐loaded SN38 NPs in tumors rather than all other organs (*p* < 0.05, compared with saline), while free Los pretreatment showed a limited effect (Figure 4C,D). In addition, quantitative detection of SN38 accumulation in tumor tissues was performed on orthotopic Panc02 and subcutaneous L3.6pl and BxPC‐3 cancer CDX models plus the PDX model using high‐performance liquid chromatography (HPLC). As suggested in Figure 4E, intratumoral SN38 concentrations were all significantly increased in the group after Los NB pretreatment rather than free Los, particularly in the PDX model, which reflects the clinical features (1.82 times vs saline, *p* < 0.01; 1.51 times vs free Los, *p* < 0.01).

Furthermore, the detailed DiR‐loaded SN38 NP distribution in tumors was analyzed by frozen sections. Even in the deep tumor parenchyma, a significant DiR signal could still be observed with Los‐LA NP pretreatment (Figure 4F). Meanwhile, DiR signal intensity at different distances from blood vessels was calculated.^[^ ^8^, ^37^ ^]^ The DiR signal was of low intensity in tumors pretreated with saline or free Los and concentrated mainly around vessels. In contrast, DiR‐loaded SN38 NPs in tumors pretreated with Los NB had a significantly stronger signal and were observed as far as 100 µm from the vessel (Figure 4G,H), indicating the great enhancement of SN38 NP penetration deep into tumor tissue induced by Los NB. In conclusion, the Los NB pretreatment had a greater enhancement on penetration and accumulation of subsequent nanoparticles, and was expected to improve the overall therapeutic efficiency of sequential treatment.

### 2.5 Los Nanoblocker Treatment Improved the Therapeutic Efficiency of SN38 NPs on Orthotopic Panc02 Tumors

**Figure 5:**
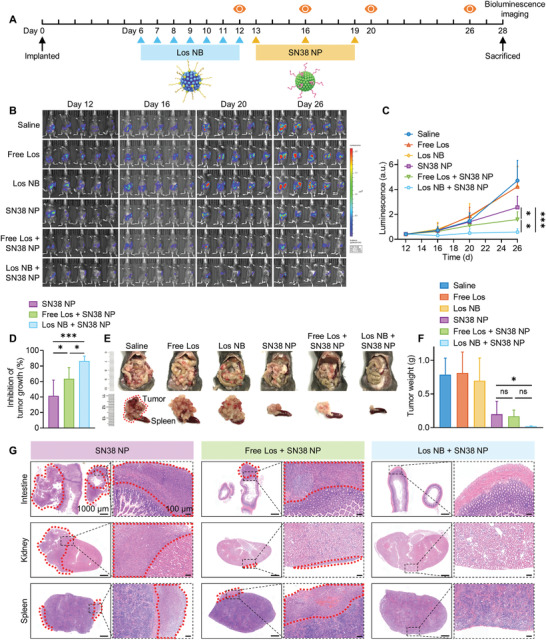
Therapeutic studies on orthotopic Panc02 cancer CDX model. A) Treatment and observation schedule. B) Bioluminescence images of orthotopic tumors. C) Quantified fluorescence intensity of the tumor sites (*n* = 5). D) The rate of tumor growth inhibition calculated from the results of fluorescence intensity on Day 26 (*n* = 5). E) Representative images of abdomen conditions and tumor tissues with spleens on Day 28. F) The average tumor weights on Day 28 (*n* = 5). G) Tumor invasion to intestine, kidney, and spleen (encircled by red dashed lines) on Day 28. Data are presented as mean ± SD. Significance was assessed by one‐way ANOVA followed by LSD post hoc test. ns, *p* > 0.05, *p < 0.05, **p < 0.01, ***p < 0.001.

Intrigued by augmented intratumoral delivery of intravenously administered SN38 NPs by Los NB pretreatment, we thus evaluated the therapeutic efficacy of the sequential combination regime to treat aggressive Panc02 orthotopic tumor. Panc02‐Luc cells were inoculated into the pancreatic tail of C57BL/6 mice for in vivo bioluminescence imaging of tumor burden. Following seven doses of Los NB (20 mg kg^−1^ losartan equivalent) posttumor implantation, cytotoxic SN38 NPs (8 mg kg^−1^ SN38 equivalent) were intravenously administered (**Figure** **5**A). Saline or free Los were also pretreated as controls. As shown in Figure 5B,C, free Los or Los NB alone did not have an antitumor effect. Treatment with SN38 NPs alone suppressed tumor growth to some extent, while sequential combination of Los NB followed by SN38 NPs produced durable tumor suppression. For example, pretreatment of Los NB greatly increased the tumor growth inhibition rate of SN38 NPs to 86.7 ± 6.2%, which was significantly different from the free Los group (63.7 ± 14.4%, *p* < 0.05) and saline group (41.5 ± 20.4%, *p* < 0.05) (Figure 5D). Moreover, measurements of tumor size and weight supported the superior efficacy of sequential combination regime (Figure 5E,F). Histological analysis also revealed that sequential combination led to extensive apoptosis and substantially reduced proliferation in tumor slices (Figure S11, Supporting Information). Collagen depletion in tumors was still observed with Masson's trichrome staining even on day 16 after the last injection of Los NB (Figure S12, Supporting Information), indicating a long‐term effect induced by nanodelivery of Los agent.

This orthotopic pancreatic tumor model enables to evaluate local tumor invasion and distant metastasis. Without cytotoxic SN38 NP treatment, invasion of cancer cells into adjacent organs was obviously observed (Figure 5G), which is similar to human pancreatic tail cancer.^[^ ^33^, ^38^ ^]^ In the mice receiving only SN38 NP treatment, local tumor invasion was inhibited but distant metastases to intestine, kidney, and spleen were still observed. Notably, Los NB followed by SN38 NPs regime inhibited invasion of Panc02 cells to neighboring organs (Figure 5G). This could be attributable to downregulated TGF‐*β* by Los NB, which ultimately inhibited epithelial–mesenchymal transition and reduced invasive and metastatic activities of tumor cells.^[^ ^39^ ^]^ These observations also suggested that in the orthotopic model, Los NB significantly enhanced the inhibitory effect of SN38 NPs on the proliferation and invasion of PDAC, and have the potential to improve the outcomes of PDAC patients. Therefore, it was verified that the Los NB pretreatment could consistently normalize the stroma‐rich TME and vascular perfusion and enhance subsequent SN38 NP penetration and accumulation deep into tumor tissues, thus promoting antitumor efficacy.

### 2.6 Los Nanoblocker Treatment Potentiates the Efficacy of Sequential SN38 NPs against PDX Model

**Figure 6:**
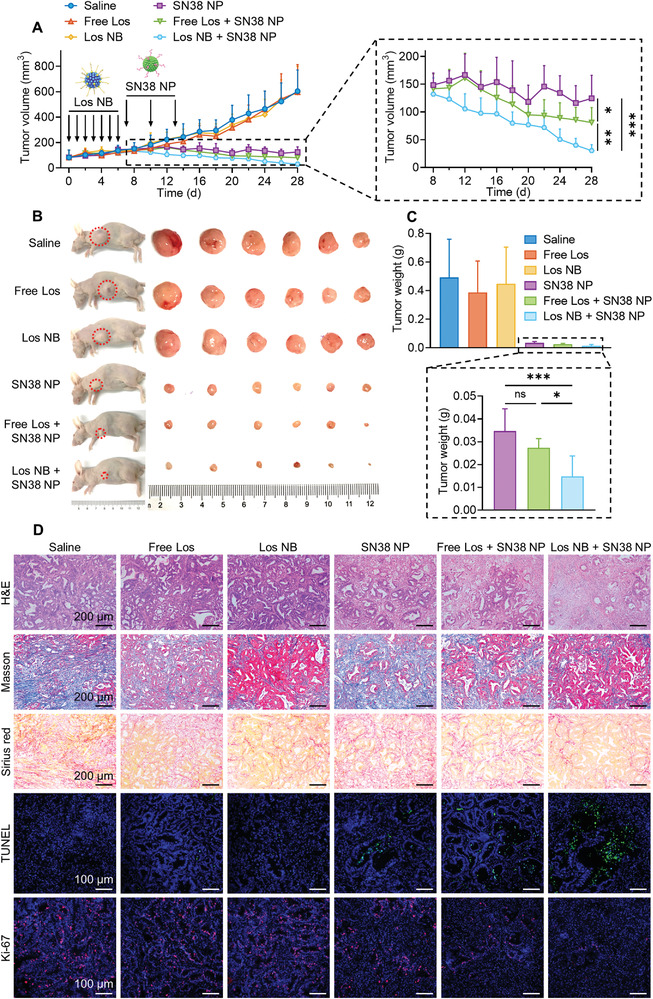
Therapeutic studies on the PDX model. A) Treatment schedule and tumor volume changes in different groups of mice (*n* = 6). B) Representative image of tumor‐bearing mice and ex vivo tumors on Day 28. C) The average tumor weights on Day 28 (*n* = 6). D) Representative hematoxylin and eosin (H&E), collagen content (Masson and Sirius red staining), TUNEL, and Ki‐67 staining images of tumor tissues on Day 28. Data are presented as mean ± SD. Significance was assessed by one‐way ANOVA followed by LSD post hoc test. ns, *p* > 0.05, *p < 0.05, **p < 0.01, ***p < 0.001.

To further assess the potential of self‐assembling antihypertension agent for clinical practice, efficacy testing was performed on a preclinical model of pancreatic PDXs. The growth of subcutaneous tumors was notably suppressed following treatment of cytotoxic SN38 NPs (**Figure** **6**A–C), which could be attributed to the high sensitivity of this PDX to topoisomerase I inhibitor. Despite this remarkable activity, Los NB further effectively improved the antitumor potential of sequentially administered SN38 NPs as compared with saline and free Los (Figure 6A–C). Masson's trichrome and Sirius red staining for collagen showed that SN38 chemotherapy alone had resulted in a high‐stromal level in TME, which has been regarded a crucial factor of chemoresistance and poor prognosis for patients.^[^ ^40^ ^]^ Again, depletion of stroma by Los NB lasting for long time was verified (Figure 6D and Figure S13, Supporting Information), which contributed to the complete eradication of tumors. Consistent with the above results, terminal deoxynucleotidyl transferase dUTP nick end labeling and Ki‐67 histopathology analyses demonstrated a high level of apoptotic rate and a low level of cancer cell proliferation upon exposure to the combinational treatment (Figure 6D and Figure S14, Supporting Information). Taken together, these satisfactory efficacies observed in the pancreatic PDX model provides potential for further clinical application of Los NB to improve chemotherapeutic efficacy.

### 2.7 Los Nanoblocker Possesses Satisfactory Biosafety

**Figure 7:**
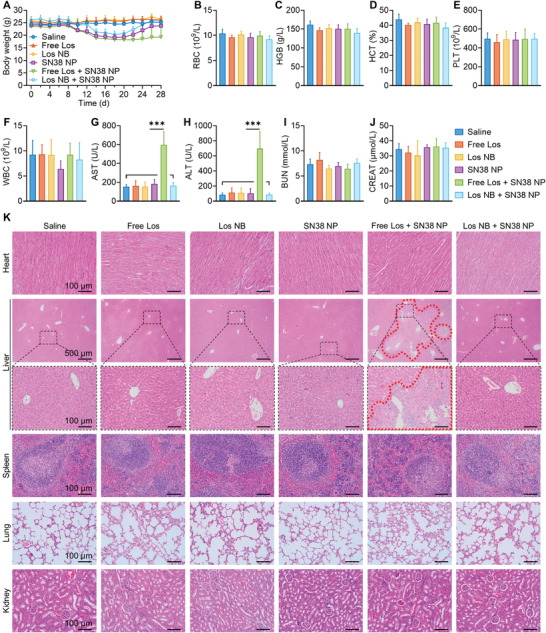
Biosafety assessment of the Los NB + SN38 NP combination in animals. A) Body weight changes in different groups of mice (*n* = 6). B–J) Analysis of biochemical parameters in whole blood, including RBC, HGB, HCT, PLT, WBC, AST, ALT, BUN, and CREAT, on Day 28 (*n* = 6). K) Representative H&E staining images of heart, liver, spleen, lung, and kidney on Day 28. Liver necrosis areas are encircled by red dashed lines. Data represent the means ± SD. Data are presented as mean ± SD. Significance was assessed by one‐way ANOVA followed by LSD post hoc test. ns, *p* > 0.05, *p < 0.05, **p < 0.01, ***p < 0.001.

Finally, to evaluate the feasibility for future clinical translation, the safety profiles of combinational therapies or monotherapies were investigated in animals. Los NB combined with or without SN38 NP did not cause noticeable changes in body weight, blood tests, or histopathology of major organs in Panc02 tumor xenograft‐bearing mice (Figures S15–S17, Supporting Information). Monotherapies with Los NB or free Los also did not show toxicities in nude mice with PDX, as evidenced by stable body weight (**Figure** **7**A). Repeated dosing of SN38 NP‐based chemotherapy resulted in a drop of mouse body weight. Unexpectedly, additive toxicity of combination of free Los with SN38 NP was observed with slowed restoration of body weight during the observation period. By contrast, the mice receiving combinatorial NP regime exhibited the loss of body weight, but rebounded to normal after cessation of treatment. Furthermore, significant increases in serum aspartate aminotransferase (AST) and alanine aminotransferase (ALT) levels were detected in mice treated with free Los followed by SN38 NP, indicating impaired liver function (Figure 7G,H), which was also supported by large areas of necrosis in the liver (encircled by red dashed lines, Figure 7K).

The reasons for hepatotoxicity caused by free Los with SN38 NP treatment were explored from different perspectives. First, losartan and Los‐LA showed no cytotoxicity against human hepatocytes HepLi‐4 and mouse hepatocytes AML12 (Figure S18A,B, Supporting Information). A combination of SN38 with losartan or Los‐LA showed no enhanced cytotoxicity (Figure S18C,D, Supporting Information). Besides, liver section staining in Figure S18E in the Supporting Information illustrated the significant hepatocyte apoptosis only in the free Los plus SN38 NP group. No liver fibrosis was observed in all groups. Moreover, BALB/c nude mice were injected a dose of saline/free losartan/Los NB following a dose of saline/SN38 NP after 24 h. At 24 h after SN38 NP injection, livers were harvested following RNA sequencing. As shown in Figure S18F, Supporting Information, the expression of UDP‐glucuronosyltransferase A1 (UGT1A) was significantly higher in the free Los plus SN38 NP group, indicating its high occupation. It has been reported that UGT1A plays an essential role in the inactivation and detoxification of SN38, and also participated in losartan metabolism.^[^ ^41^ ^]^ Therefore, we considered that free losartan rapidly occupied UGT1A in the liver and inhibited the metabolism of the following SN38 NP. In contrast, Los NB owned prolonging blood circulation and sustained‐release capacity (Figure 2G), thus might have a limited occupation on UGT1A and ensured the timely detoxification of SN38 NP. Since the metabolic capacity of UGT1A varied from different subtypes and different species, the details deserve further investigation.^[^ ^41b^ ^]^

Encouragingly, combination of Los NB and sequential SN38 NP therapy did not present obvious side effects via routine blood and biochemistry tests of liver and kidney function (Figure 7B–J), and showed no significant organ injury (Figure 7K). These detailed in vivo data provided compelling evidence that Los NB can be safely administered via intravenous injection or combined with other chemotherapies.

## 3 Conclusion

In this study, losartan, a widely clinically approved antihypertension drug, was explored to remodel the dense‐stromal TME and enhance nanodrug chemotherapy efficacy in pancreatic cancer. A new losartan‐loaded nanoparticle was designed via small‐molecule assembling strategy with high EE/DL, good stability, and sustained‐release capacity. Compared with free losartan, Los NB showed more potent effects on tumor stroma modulation and TME remodeling in PDAC. With Los NB pretreatment increasing tumor vessel perfusion and depleting the dense stromal barrier, the accumulation and penetration of sequential SN38 NPs deep into tumor tissues were significantly enhanced. The overall therapeutic efficacy of SN38 NPs was remarkable increased by Los NB pretreatment, and the satisfactory biosafety of Los‐NB was verified. Given these results, the losartan‐loaded nanoparticle system might provide a promising strategy for stroma‐rich PDAC treatment.

## 4 Experimental Section

### Synthesis of Losartan Prodrugs

Five losartan‐fatty acid conjugates were synthesized and characterized by ^1^H NMR and MS. The synthetic protocols are provided in the Supporting Information.

### Cell Lines, Animals, and Antibodies

Human pancreatic adenocarcinoma cell lines (L3.6pl and BxPC‐3), murine pancreatic adenocarcinoma cell lines (Panc02), murine embryonic fibroblasts (NIH‐3T3), and murine hepatocytes (AML12) were purchased from the Cell Bank of the Chinese Academy of Sciences (Shanghai, China). Human hepatocytes (HepLi‐4) were a generous gift from Lanjuan Li's laboratory.^[^ ^42^ ^]^ L3.6pl, BxPC‐3, and Panc02 cells were cultured in RPMI‐1640 medium with 10% FBS. NIH‐3T3 and HepLi‐4 cells were cultured in Dulbecco's modified Eagle medium with 10% FBS. AML12 cells were cultured in specialized medium (CM‐0602, Procell, China). All cells were cultured at 37 °C in humid air with 5% CO_2_. SD rats (male, 8 weeks old), C57BL/6, and BALB/c nude mice (male, 5 weeks old) were purchased from the Shanghai Experimental Animal Center, Chinese Academy of Science, and housed at the Zhejiang Academy of Medical Sciences. All animal experiments followed the National Institute Guide for the Care and Use of Laboratory Animals. The experimental protocols were approved by the Ethics Committee of the First Affiliated Hospital, School of Medicine, Zhejiang University (Reference Number: 2019 No. 1218). Antibodies used for Western blot and immunostaining staining are listed in Table S1, Supporting Information.

### Preparation of Self‐Assembled Losartan‐Loaded Nanoparticles

Los‐LA conjugate and DSPE‐PEG_2k_ were dissolved in DMSO separately and premixed at a ratio of 10:1 w/w. DMSO solution (1 mL) of Los‐LA prodrug (16.2 mg) and DSPE‐PEG_2k_ (1.8 mg) was slowly injected into DI water (9 mL) under ultrasound. Then, DMSO was removed by dialysis against DI water.

### Establishment of the PDX Subcutaneous Mouse Model

The patient was fully informed and signed a written informed patient consent form. The Ethics Committee of the First Affiliated Hospital, Zhejiang University School of Medicine, approved the experimental protocol. Fresh human PDAC tumor tissue was surgically resected from the PDAC patient, cut into ≈1 mm^3^ pieces, and subcutaneously implanted into the flank area of BALB/c nude mice. PDX tumors were subcutaneously passaged into other nude mice, and third‐generation tumor‐bearing mice were used for experiments.^[^ ^24^ ^]^ Mice with a tumor volume of ≈100 mm^3^ were randomly divided into three groups (*n* = 5) and intravenously injected with free Los or Los NB at a 20 mg kg^−1^ losartan equivalent dose or equal volume of saline as a control daily for 7 consecutive days. 24 h after the last injection, the tumor tissues were harvested for histological and collagen content analysis.

### Establishment of the Panc02 Orthotopic Pancreatic Cancer Mouse Model

To establish bioluminescent orthotopic pancreatic cancer models, Panc02 cells were first transfected with luciferase and puromycin resistance genes and selected using 2 µg mL^−1^ puromycin for stable expression of luciferase. Using an insulin‐gauge syringe, 20 µL of phosphate‐buffered saline (PBS)/Matrigel (1:1, v/v) containing 2 × 10^5^ Panc02 cells was injected into the tail of the pancreas of C57BL/6 mice. After 6 days, mice were randomly divided into three groups (*n* = 5). The protocol of drug administration was the same as that used for PDX mouse models. 24 h after the last injection, the tumor tissues were harvested for histological, collagen content, and tumor‐infiltrating immunologic factor analysis.

### Sircol Soluble Collagen Assay and Solid Stress Measurement

The tumor collagen content was measured using Sircol Collagen Assay Kit (Biocolor, UK) according to the manufacturer's instructions. PDX mice with tumors that reached a size of ≈1 cm in diameter were selected for solid stress measurement. The protocol of grouping and drug administration was the same as described above. 24 h after the last injection, the tumor tissues were harvested, washed with Hank's buffer, and measured using a caliper. Solid stress was measured using the tumor opening technique.^[^ ^30^ ^]^ The tumor was cut along its longest axis (≈80% of its shortest diameter) and then soaked in Hank's buffer for 10 min to diminish any transient, poroelastic responses. Next, the width of the opening at the surface of the tumor at the middle of the cut was measured. Finally, the width of the opening was divided by the diameter perpendicular to the cut to calculate the solid stress.

### Intravital Imaging of Nanoparticles in Panc02 Orthotopic Models

6 days after tumor inoculation, mice were randomly divided into three groups (*n* = 3) and intravenously injected with a solution containing free Los or Los NB at a 20 mg kg^−1^ losartan equivalent dose or an equal volume of saline as a control, respectively, daily for 7 consecutive days. 1 h after the last injection, mice were intraperitoneally injected with D‐luciferin (75 mg kg^−1^) (YEASEN, 40902ES03) and subjected to bioluminescence imaging in vivo using IVIS Lumina LT (PerkinElmer) for tumor size quantification. OligoCL_46_‐SN38 conjugate‐loaded nanoparticles (SN38 NPs) were employed as a subsequent model nanoparticle, which were previously published.^[^ ^14^ ^]^ To track the in vivo distribution, a lipophilic near‐infrared fluorescence probe, DiR, was coassembled into SN38 NPs. 24 h after the last injection, DiR‐loaded SN38 NPs were intravenously injected into mice (1 mg kg^−1^ DiR equivalent concentration). The in vivo distribution of DiR‐loaded SN38 NPs was quantitatively visualized using IVIS Lumina LT at predetermined time points. At 24 h, the mice were sacrificed, and tumors and major organs were collected for ex vivo imaging. The excitation wavelength was set at 745 nm, and the emission wavelength was set at 780 nm.

### SN38 Distribution in Tumor Tissues

In addition to the PDX subcutaneous models described above, subcutaneous L3.6pl and BxPC‐3 PDAC models by inoculation of 2 × 10^6^ L3.6pl or BxPC‐3 cells in PBS (100 µL) into the right flank of 5 weeks old nude mice were also established. When the tumor volume reached ≈100 mm^3^, mice were selected and randomly divided into three groups (*n* = 5), intravenously injected with solution containing free Los or Los NB at 20 mg kg^−1^ losartan equivalent dose or equal volume of saline as a control, respectively, daily for 7 consecutive days. 24 h after the last injection, SN38 NPs were intravenously injected into mice at an SN38 dose of 8 mg kg^−1^. After 24 h, the mice were sacrificed, and tumor tissues were collected and homogenized. SN38 in tumor tissues was extracted by acetonitrile and the concentration was determined by HPLC.

### In Vivo Antitumor Efficacy Study

6 days after Panc02 cell inoculation, mice were randomly divided into six groups (*n* = 5) and treated as follows: saline followed by saline, free Los followed by saline, Los NB followed by saline, saline followed by SN38 NPs, free Los followed by SN38 NPs, and Los NB followed by SN38 NPs. For saline/free Los/Los NB treatment, mice were injected with an equal volume of saline, a solution containing free Los or Los NB (20 mg kg^−1^ losartan equivalent dose), daily for 7 consecutive days. For subsequent saline/SN38 NP treatment, 24 h after the last injection, mice were intravenously injected with an equal volume of saline or solution containing SN38 NPs (8 mg kg^−1^ SN38 equivalent dose), once every 3 days three times. Bioluminescence imaging in vivo was used to quantify tumor growth. After Day 28, mice were sacrificed, and the tumors were harvested for weight and histological analysis.

PDX mice with a tumor volume of 100 mm^3^ were randomly divided into six groups (*n* = 6). The protocol of drug administration was the same as described above. The tumor size was measured using a caliper, and the body weight of the mice was recorded every 2 days. The tumor volume was calculated as 1/2 × length × width^2^. On Day 28, the mice were sacrificed, and the tumors were harvested for weight and histological analysis.

### Blood Routine and Biochemistry Analysis

Whole blood samples were obtained from the mice before sacrifice. Blood cell count (RBC), red hemoglobin count (HGB), hematocrit (HCT), platelet count (PLT), and white blood cell count (WBC) were analyzed. The concentrations of aspartate aminotransferase (AST), alanine aminotransferase (ALT), blood urea nitrogen (BUN), and creatinine (CREAT) in the serum were detected as indicators of hepatorenal function.

### Statistical Analysis

Experiments were repeated a minimum of three times. The quantitative data were presented as means ± standard deviation (SD). Student's *t* test and one‐way analysis of variance (ANOVA) test followed by least significant difference (LSD) post hoc test were performed to assess the statistical significance between two groups and among multiple groups, respectively. Differences with *p* < 0.05 defined statistically significant (*p < 0.05, **p < 0.01, ***p < 0.001). SPSS software (SPSS Inc., USA) was used for data analysis.

## Conflict of Interest

The authors declare no conflict of interest.

## Supporting information

Supporting Information

## Data Availability

The data that support the findings of this study are available from the corresponding author upon reasonable request.
