# Supplementary material for: Antihypertension Nanoblockers Increase Intratumoral Perfusion of Sequential Cytotoxic Nanoparticles to Enhance Chemotherapy Efficacy against Pancreatic Cancer
Source: Adv Sci (Weinh). 2022 Aug 26;9(29):2201931. doi: 10.1002/advs.202201931 (PMC9561769; doi:10.1002/advs.202201931)
Supplement: Supplementary file 1 — Supporting Information [file ADVS-9-2201931-s001.pdf]

ADVANCED SCIENCE
Open Access

Supporting Information

for *Adv. Sci.*, DOI 10.1002/advs.202201931

Antihypertension Nanoblockers Increase Intratumoral Perfusion of Sequential Cytotoxic Nanoparticles to Enhance Chemotherapy Efficacy against Pancreatic Cancer

Suchen Bian, Haijiang Dong, Long Zhao, Zequn Li, Jian Chen, Xingxin Zhu, Nasha Qiu, Xing Jia, Wenfeng Song, Zekuan Li, Shusen Zheng, Hangxiang Wang* and Penghong Song**

Supporting Information for

Antihypertension nanoblockers increase intratumoral perfusion of sequential cytotoxic nanoparticles to enhance chemotherapy efficacy against pancreatic cancer

Suchen Bian,[a,b,c,d,‡] Haijiang Dong,[a,b,c,d,‡] Long Zhao,[a,b,c,d] Zequn Li,[a,b,c,d] Jian Chen,[e] Xingxin Zhu,[a,b,c,d] Nasha Qiu,[e] Xing Jia,[a,b,c,d] Wenfeng Song,[a,b,c,d] Zekuan Li,[a,b,c,d] Shusen Zheng,[a,b,c,d,*] Hangxiang Wang,[a,b,c,d,*] Penghong Song [a,b,c,d,*]

[a] Division of Hepatobiliary and Pancreatic Surgery, Department of Surgery, The First Affiliated Hospital, Zhejiang University School of Medicine, Hangzhou 310003, China.

[b] NHC Key Laboratory of Combined Multi-organ Transplantation, Hangzhou 310003, China.

[c] Key Laboratory of the diagnosis and treatment of organ Transplantation, Research Unit of Collaborative Diagnosis and Treatment for Hepatobiliary and Pancreatic Cancer, Chinese Academy of Medical Sciences (2019RU019), Hangzhou 310003, China.

[d] Key Laboratory of Organ Transplantation, Research Center for Diagnosis and Treatment of Hepatobiliary Diseases, Zhejiang Province, Hangzhou 310003, China.

[e] Department of Hepatobiliary and Pancreatic Surgery, The Center for Integrated Oncology and Precision Medicine, Affiliated Hangzhou First People's Hospital Zhejiang University School of Medicine, Hangzhou 310006, China.

Corresponding Authors

Penghong Song, The First Affiliated Hospital, Zhejiang University School of Medicine; 79, Qingchun Road, Hangzhou 310003, China. Tel: 86-571-87236466; Fax: 86-571-87236466; E-mail: songpenghong@zju.edu.cn.

Hangxiang Wang, The First Affiliated Hospital, Zhejiang University School of Medicine; 79, Qingchun Road, Hangzhou 310003, China. Tel: 86-571-87236466; Fax: 86-571-87236466; E-mail: wanghx@zju.edu.cn.

Shusen Zheng, The First Affiliated Hospital, Zhejiang University School of Medicine; 79, Qingchun Road, Hangzhou 310003, China. Tel: 86-571-87236466; Fax: 86-571-87236466; E-mail: shusenzheng@zju.edu.cn.

‡ These authors contributed equally to this work.

Supplementary Materials and Methods

Materials

Losartan, butyric acid, decanoic acid, oleic acid, linoleic acid and linolenic acid were purchased from Tokyo Chemical Industry Co., Ltd. (Shanghai, China). 1,2-Distearoyl-*sn*-glycero-3-phosphoethanolamine-*N*-[methoxy (polyethylene glycol) 2000] (DSPE-PEG_{2000}) was purchased from A.V.T. Pharmaceutical Co., Ltd. (Shanghai, China). All other compounds and solvents were purchased from J&K Chemical (Shanghai, China).

Synthesis of losartan prodrugs

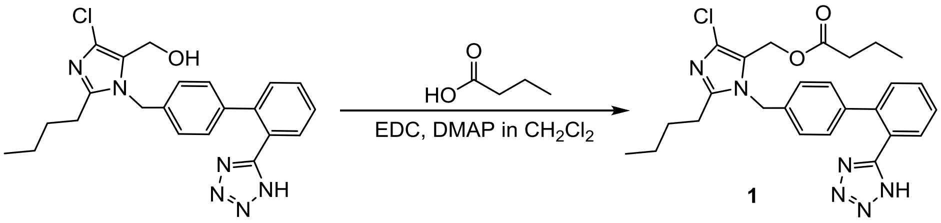

1

Scheme S1. Synthetic scheme of 1.

To a solution of losartan (100.0 mg, 0.236 mmol) and butyric acid (20.8 mg, 0.236 mmol) in 3 mL of Methylene chloride (DCM) was added 1-ethyl-3-(3-(dimethylamino)propyl) carbodiimide (EDC; 90.7 mg, 0.472 mmol) and 4-dimethylaminopyridine (DMAP; 43.3 mg, 0.354 mmol). The reaction solution was stirred and heated at 70 ℃ for 12 h and then washed with saturated sodium chloride, dried over anhydrous sodium sulfate and evaporated under vacuum. Column chromatography on silica gel in dichloromethane/ methanol (80:1) as the mobile phase was used to purify the target compound 1 (yellow oily liquid, 47.1 mg, 40.4%).

^{1}H NMR (400 MHz, Chloroform-*d*) δ 7.86 (d, *J* = 7.6 Hz, 1H), 7.55 (dt, *J* = 31.0, 7.6 Hz, 2H), 7.38 (d, *J* = 7.6 Hz, 1H), 7.03 (d, *J* = 7.7 Hz, 2H), 6.77 (d, *J* = 7.8 Hz, 2H), 5.09 (s, 2H), 4.78 (s, 2H), 2.46 (t, *J* = 7.8 Hz, 2H), 2.11 (t, *J* = 7.5 Hz, 2H), 1.61 (t, *J* = 7.8 Hz, 2H), 1.51 (q, *J* = 7.4 Hz, 2H), 1.32 (d, *J* = 7.8 Hz, 2H), 0.89 – 0.83 (m, 6H).

HR-ESI Qq-LTMS: calcd for $[C_{26}H_{29}ClN_6O_2]^+$ $[M+H]^+$ = 493.2119; obsd 493.2123.

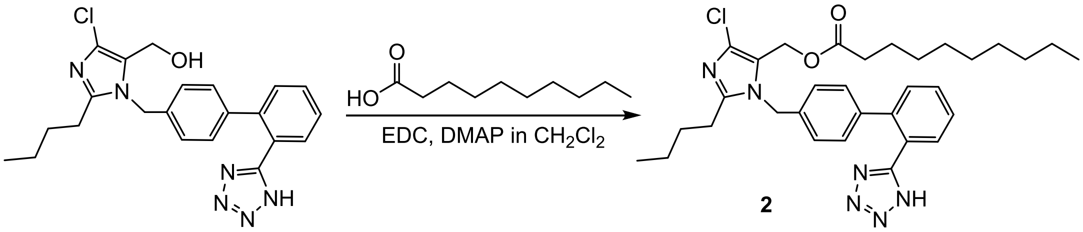

Scheme S2. Synthetic scheme of 2.

To a solution of losartan (100.0 mg, 0.236 mmol) and decanoic acid (40.7 mg, 0.236 mmol) in 3 mL of DCM was added EDC (90.7 mg, 0.472 mmol) and DMAP (43.3 mg, 0.354 mmol). The reaction solution was stirred and heated at 70 ℃ for 12 h and then washed with saturated sodium chloride, dried over anhydrous sodium sulfate and evaporated under vacuum. Column chromatography on silica gel in dichloromethane/ methanol (80:1) as the mobile phase was used to purify the target compound 2 (yellow oily liquid, 52.7 mg, 38.6%).

^{1}H NMR (400 MHz, Chloroform-*d*) δ 7.90 (d, *J* = 7.6 Hz, 1H), 7.57 (dt, *J* = 26.3, 7.5 Hz, 2H), 7.41 (d, *J* = 7.6 Hz, 1H), 7.10 (d, *J* = 7.8 Hz, 2H), 6.80 (d, *J* = 7.8 Hz, 2H), 5.13 (s, 2H), 4.83 (s, 2H), 2.42 (t, *J* = 7.8 Hz, 2H), 2.09 (t, *J* = 7.6 Hz, 2H), 1.59 (t, *J* = 7.8 Hz, 2H), 1.46 (q, *J* = 7.4 Hz, 2H), 1.37 – 1.19 (m, 14H), 0.87 (td, *J* = 7.2, 3.2 Hz, 6H).

HR-ESI Qq-LTMS: calcd for $[C_{32}H_{41}ClN_6O_2]^+$ $[M+H]^+$ = 577.3058; obsd 577.3057.

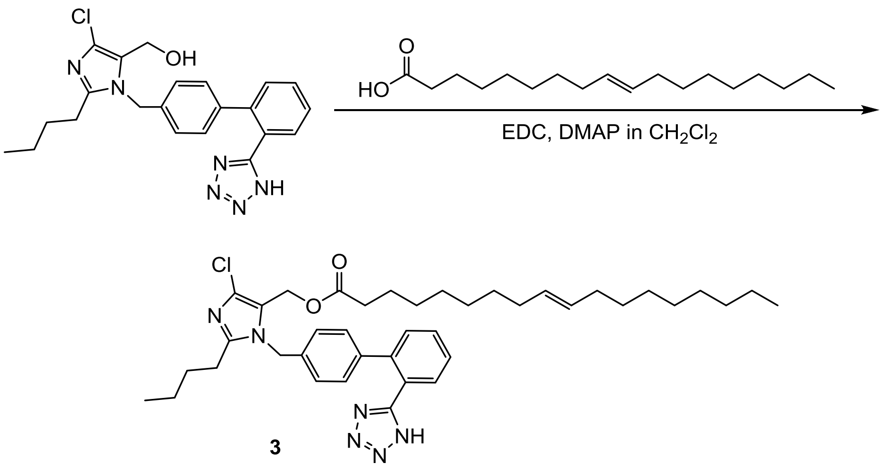

Scheme S3. Synthetic scheme of 3.

To a solution of losartan (100.0 mg, 0.236 mmol) and oleic acid (66.8 mg, 0.236 mmol) in 3 mL of DCM was added EDC (90.7 mg, 0.472 mmol) and DMAP (43.3 mg, 0.354 mmol). The reaction solution was stirred and heated at 70 ℃ for 12 h and then washed with saturated sodium chloride, dried over anhydrous sodium sulfate and evaporated under vacuum. Column chromatography on silica gel in dichloromethane/ methanol (80:1) as the mobile phase was used to purify the target compound 3 (yellow oily liquid, 68.2 mg, 42.0%).

^{1}H NMR (400 MHz, Chloroform-*d*) δ 7.96 (d, *J* = 7.6 Hz, 1H), 7.57 (dt, *J* = 23.7, 7.6 Hz, 2H), 7.41 (d, *J* = 7.5 Hz, 1H), 7.12 (d, *J* = 7.8 Hz, 2H), 6.83 (d, *J* = 7.8 Hz, 2H), 5.32 (q, *J* = 6.4 Hz, 2H), 5.14 (s, 2H), 4.83 (s, 2H), 2.45 (t, *J* = 7.9 Hz, 2H), 2.10 (t, *J* = 7.6 Hz, 2H), 2.02 – 1.95 (m, 4H), 1.62 (q, *J* = 7.7 Hz, 2H), 1.47 (p, *J* = 7.1 Hz, 2H), 1.36 – 1.22 (m, 22H), 0.88 (td, *J* = 7.2, 3.1 Hz, 6H).

HR-ESI Qq-LTMS: calcd for $[C_{40}H_{55}ClN_6O_2]^+$ $[M+H]^+$ = 687.4153; obsd 687.4157.

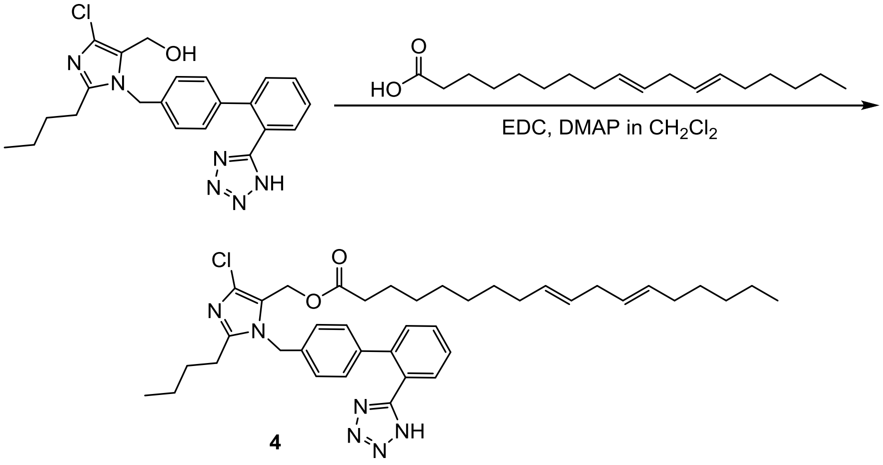

4

Scheme S4. Synthetic scheme of 4.

To a solution of Losartan (100.0 mg, 0.236 mmol) and linoleic acid (66.3 mg, 0.236 mmol) in 3 mL of DCM was added EDC (90.7 mg, 0.472 mmol) and DMAP (43.3 mg, 0.354 mmol). The reaction solution was stirred and heated at 70 ℃ for 12 h and then washed with saturated sodium chloride, dried over anhydrous sodium sulfate and evaporated under vacuum. Column chromatography on silica gel in dichloromethane/ methanol (80:1) as the mobile phase was used to purify the target compound 4 (yellow oily liquid, 70.1 mg, 47.3%).

^{1}H NMR (400 MHz, Chloroform-*d*) δ 7.93 (dd, *J* = 7.6, 1.5 Hz, 1H), 7.58 (dtd, *J* = 24.2, 7.6, 1.5 Hz, 2H), 7.41 (dd, *J* = 7.5, 1.4 Hz, 1H), 7.16 – 7.08 (m, 2H), 6.80 (d, *J* = 8.0 Hz, 2H), 5.40 – 5.30 (m, 4H), 5.14 (s, 2H), 4.84 (s, 2H), 2.75 (t, *J* = 6.2 Hz, 2H), 2.44 – 2.36 (m, 2H), 2.11 – 1.98 (m, 6H), 1.62 – 1.54 (m, 2H), 1.45 (q, *J* = 7.3 Hz, 2H), 1.36 – 1.18 (m, 16H), 0.88 (td, *J* = 7.1, 2.7 Hz, 6H).

HR-ESI Qq-LTMS: calcd for $[C_{40}H_{53}ClN_6O_2]^+$ $[M+H]^+$ = 685.3997; obsd 685.4014.

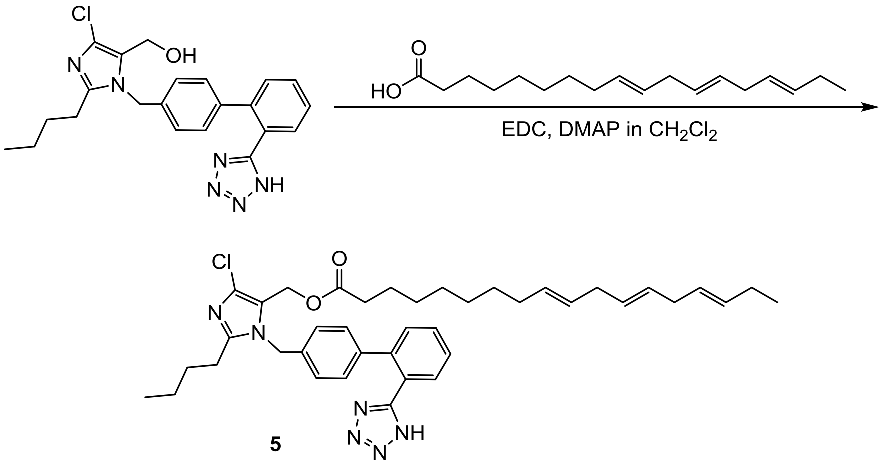

Scheme S5. Synthetic scheme of 5.

To a solution of Losartan (100.0 mg, 0.236 mmol) and linolenic acid (65.8 mg, 0.236 mmol) in 3 mL of DCM was added EDC (90.7 mg, 0.472 mmol) and DMAP (43.3 mg, 0.354 mmol). The reaction solution was stirred and heated at 70 ℃ for 12 h and then washed with saturated sodium chloride, dried over anhydrous sodium sulfate and evaporated under vacuum. Column chromatography on silica gel in dichloromethane/ methanol (80:1) as the mobile phase was used to purify the target compound 4 (yellow oily liquid, 65.5 mg, 40.5%).

^{1}H NMR (400 MHz, Chloroform-*d*) δ 7.94 (d, *J* = 7.6 Hz, 1H), 7.57 (dt, *J* = 24.4, 7.5 Hz, 2H), 7.40 (d, *J* = 7.5 Hz, 1H), 7.11 (d, *J* = 7.8 Hz, 2H), 6.83 (d, *J* = 7.8 Hz, 2H), 5.41 – 5.29 (m, 6H), 5.13 (s, 2H), 4.83 (s, 2H), 2.79 (q, *J* = 5.3, 4.9 Hz, 4H), 2.45 (t, *J* = 7.9 Hz, 2H), 2.06 (dt, *J* = 23.4, 7.4 Hz, 6H), 1.61 (t, *J* = 7.8 Hz, 2H), 1.47 (t, *J* = 7.3 Hz, 2H), 1.39 – 1.22 (m, 10H), 0.97 (t, *J* = 7.5 Hz, 3H), 0.88 (t, *J* = 7.3 Hz, 3H).

HR-ESI Qq-LTMS: calcd for $[C_{40}H_{51}ClN_6O_2]^+$ $[M+H]^+$ = 683.3840; obsd 683.3799.

Determination of EE and DL

The encapsulation efficiency (EE) and drug loading (DL) contents of losartan in nanoparticles were determined by high-performance liquid chromatography (HPLC). The nanoparticle solutions were centrifuged at 4000 rpm for 5 min. Then, the supernatants were collected for Los-LA determination by HPLC. HPLC was performed using a Hitachi Chromaster 5000 system with a YMC-Pack ODS-A column (5 μm, 250 × 4.6 mm) at a flow rate of 1.0 mL/min. UV detection was performed at 220 nm. All of the runs used linear gradients of acetonitrile (solvent A) and water (solvent B) containing 0.1% trifluoroacetic acid (TFA). The EE and DL values were calculated according to Equations (1) and (2):

EE (%) = $W_{\text{Los in NPs}} / W_{\text{fed}} \times 100\%$ (1)

DL (%) = $W_{\text{Los in NPs}} / W_{\text{total}} \times 100\%$ (2)

$W_{\text{Los in NPs}}$, W_{fed}, and W_{total} represent the weights of losartan formulated into nanoparticles, the initial prodrugs fed for encapsulation, and the total amount of nanoparticles, respectively.

Characterization of nanoparticles by DLS

The hydrodynamic diameters of the nanoparticles were characterized through dynamic light scattering (DLS) measurements on a Malvern Nano-ZS90 instrument (Malvern, UK) at 25 ℃. Each sample was measured three times.

Morphological study of nanoparticles by TEM

The sample solution of Los-LA prodrug-loaded NPs at a concentration of 0.5 mg/mL (losartan equivalent) was dropped onto a 400-mesh copper grid coated with carbon. After 5 min of deposition, the surface liquid was removed with filter papers. Samples were stained with 2 wt% aqueous uranyl acetate solution for 1 min of positive staining and air-dried. The morphology of the nanoparticles was characterized using TECNAL 10 (Philips) at an acceleration voltage of 80 kV.

***In vitro* losartan release kinetics**

Three milliliters of solutions containing free losartan@DSPE-PEG_{2000} micelles or Los-LA prodrug-loaded nanoparticles (0.1 mg/mL losartan equivalent concentration) in phosphate-buffered saline (PBS) (PBS and PBS containing 30 U/mL or 60 U/mL porcine liver esterase (PLE))

were loaded into dialysis bags (spectrum, molecular weight cutoff of 7 kDa) and dialyzed against 20 mL of releasing medium (PBS, pH 7.4, 0.4% Tween 80). The dialysis bags were continuously shaken in an orbital shaker (100 rpm) at 37 ℃. At predetermined time points, the releasing medium was collected to evaluate released losartan and Los-LA by HPLC.

Pharmacokinetic analysis of Los NB

Pharmacokinetic analysis was performed on Sprague-Dawley (SD) rats (male, 8 weeks old, n = 3/group). A single dose of free losartan or Los NB (20 mg/kg losartan equivalent dose) was intravenously injected via the tail vein. At predetermined time points, blood samples were taken following centrifuging at 3000 rpm for 10 min to gain plasma samples. The drugs were extracted by acetonitrile and the concentration was determined by liquid chromatography mass spectrometry (LCMS).

In vitro cytotoxicity analysis

Cells were planted in 96-well plates with the density of 4000-6000 cells per well. After 12 h, Drugs were added to the cells at various concentrations and incubated for an additional 24 h. Cell viability was investigated by CCK-8 assay. CCK-8 solution was added in each well (1:10, v/v) and continuously incubated for 1 h. The absorbance was measured at 450 nm using a microplate reader (Multiskan FC, Thermo Fisher Scientific). Cell viability (%) = absorbance of each well / absorbance of control well × 100%.

Cellular esterase activity analysis

Esterase activity of Panc02 and NIH-3T3 cells was measured with fluorescein diacetate (FDA), a cell-permeating esterase substrate. FDA was dissolved in DMSO at 10 mg/mL as a stock solution and diluted by PBS to 1 mg/mL as a working solution. Cells were planted in 96-well plates with the density of 1×10^4 cells per well. After 12 h, FDA working solution was added in each well (20 μg/mL final concentration) and the cells were incubated in dark. FITC fluorescence intensity was detected at frequent intervals using a microplate reader (Varioskan LUX, Thermo Fisher Scientific) (Ex = 480 nm, Em = 530 nm). For fluorescence imaging of esterase activity, cells were planted in confocal dishes with the density of 1×10^5 cells per dish. After 12 h, FDA working solution was added (5 μg/mL final concentration) and incubated for 30min in dark before observation.

Enzyme-linked immunosorbent assay (ELISA)

Panc02 and NIH-3T3 cells were planted in 12-well plates with the density of 1×10^5 cells per well. After 12 h, culture medium was removed and replaced with serum-free medium (to decrease the cross-reactivity from FBS in ELISA), and cells were incubated with PBS or the same concentration of free losartan/Los-LA/Los NB (20 μM losartan equivalent) for 24 h. The supernatants were collected and the secretion level of TGF-β was determined through an ELISA kit (No. KE10005, Proteintech, China).

Immunofluorescence

Panc02 and NIH-3T3 cells were planted in confocal dishes with the density of 5×10^4 cells per dish. After 12 h, cells were incubated with PBS or the same concentration of free losartan/Los-LA/Los NB (20 μM losartan equivalent) for 24 h. After being fixed in 4% PFA, Panc02 cells were stained with TGF-β antibody, and NIH-3T3 cells were stained with α-smooth muscle actin antibody (α-SMA) and fibronectin antibody. Cells were examined by optical microscope.

Multiplex tumor-infiltrating immunologic factors assay

Total protein of harvested Panc02 tumor tissue was extracted after ultrasonic grinding in PBS and centrifugation and was evaluated with a bicinchoninic acid (BCA) protein assay kit (Fdbio Science, Hangzhou, China). The tumor-infiltrating immunologic factors were evaluated using a LEGENDplex multiple assays kit (BioLegend, USA) according to the manufacturer's instructions.

Western blot analysis

Total protein of harvested tumor tissue was extracted after homogenization in RIPA buffer and centrifugation and was determined with a BCA protein assay kit. After separation on SDS-polyacrylamide gels, the proteins were transferred onto a PVDF membrane (Millipore, USA). The membrane was blocked with 5% bovine serum albumin for 1 hour and then incubated with primary antibodies at 4 ℃ overnight. The next day, the membrane was incubated with secondary antibodies for 1 hour at room temperature. Signals were detected using an ECL kit (Fdbio Science, Hangzhou, China). β-actin was used as a loading control.

Histological analysis

To evaluated the effect of Los NB on the tumor microenvironment, harvested tumor tissues were fixed in 4% PFA, embedded in paraffin, sectioned into 5 μm thick sections, and stained with α-smooth muscle actin antibody (α-SMA), fibronectin and hypoxia inducible factor-1α (HIF-1α)

antibodies. Slides were examined by optical microscope.

To evaluate the effect of Los NB on tumor vessel perfusion, mice were intravenously injected with DyLight 488 Lycopersicon Esculentum (Tomato) Lectin (Vectorlab, USA) 5 minutes prior to sacrifice. Harvested tumor tissues were embedded and frozen in OCT, sectioned into 5 μm thick sections, and stained with CD31 antibody. Slides were examined by confocal microscopy (Olympus, FV3000, Japan). Tumor vessel perfusion was quantitatively evaluated by calculating the percentage of perfused vessels (CD31 colocalized with lectin) among total vessels (total CD31).

To detect the distribution of DiR-loaded SN38 NPs in tumors, tumor tissues were prepared into 5 μm frozen sections, stained with CD31 antibody, and examined by confocal microscopy.

To investigate the stroma, apoptosis and proliferation levels in tumors after antitumor treatment, sections were analyzed by hematoxylin and eosin (H&E), Masson's trichrome staining, TdT-mediated dUTP nick end-labeling (TUNEL) and Ki-67 staining.

Five randomly chosen visual fields were quantitatively analyzed using ImageJ software.

RNA-sequencing Analysis

RNA sequencing was performed on the liver of mice undergoing different treatment. BALB/c nude mice were injected a dose of saline/free losartan/Los NB (20 mg/kg losartan equivalent) following a dose of saline/SN38 NP (8 mg/kg SN38 equivalent) after 24 h. At 24 h after SN38 NP injection, livers were harvested. Total RNA was extracted using Trizol reagent kit (Invitrogen, USA) according to the manufacturer's protocol. RNA quality was assessed on an Agilent 2100 Bioanalyzer (Agilent Technologies, USA) and checked using RNase free agarose gel electrophoresis. After total RNA was extracted, eukaryotic mRNA was enriched by Oligo(dT) beads. Then the enriched mRNA was fragmented into short fragments using fragmentation buffer and reversly transcribed into cDNA by using NEBNext Ultra RNA Library Prep Kit for Illumina (NEB #7530, New England Biolabs, USA). The purified double-stranded cDNA fragments were end repaired, A base added, and ligated to Illumina sequencing adapters. The ligation reaction was purified with the AMPure XP Beads. Ligated fragments were subjected to size selection by agarose gel electrophoresis and polymerase chain reaction (PCR) amplified. The resulting cDNA library

was sequenced using Illumina Novaseq6000 by Gene Denovo Biotechnology Co. (Guangzhou, China).

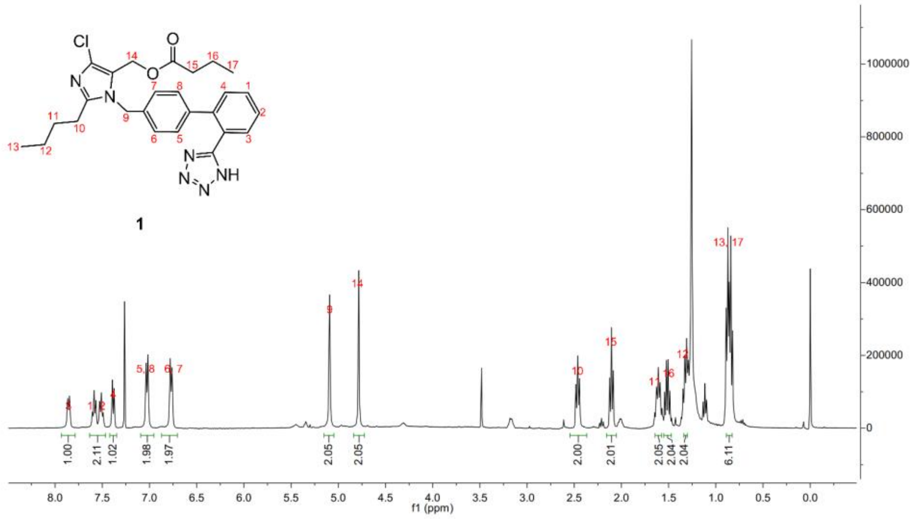
Cl
N
O
NH
1
13, 17
f1 (ppm)
1000000
800000
600000
400000
200000
0
1.00 2.11 1.02 1.98 1.97 2.05 2.05 2.00 2.01 2.05 2.04 2.04 6.11

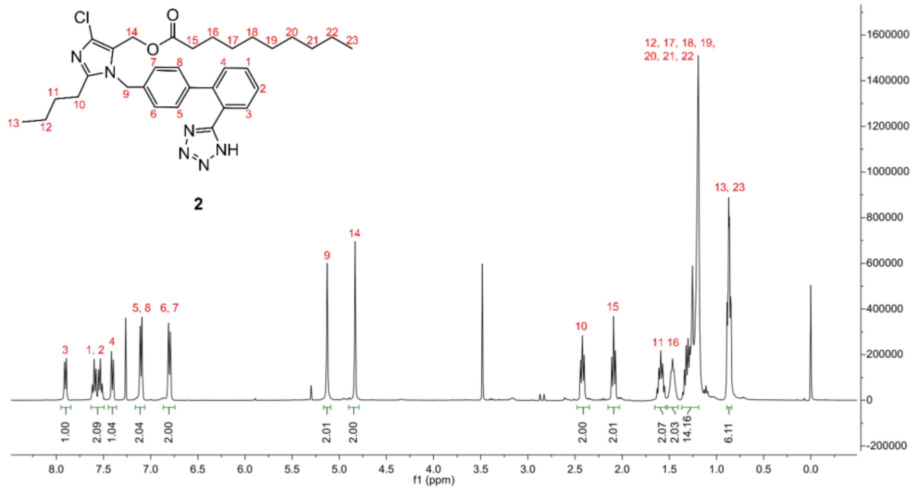
Cl
N
O
NH
2
12, 17, 18, 19,
20, 21, 22
13, 23
9
14
5, 8
6, 7
3
1, 2
4
10
15
11 16
f1 (ppm)
1600000
1400000
1200000
1000000
800000
600000
400000
200000
0
-200000
1.00 2.09 1.04 2.04 2.00 2.01 2.00 2.00 2.01 2.07 2.03 14.16 6.11

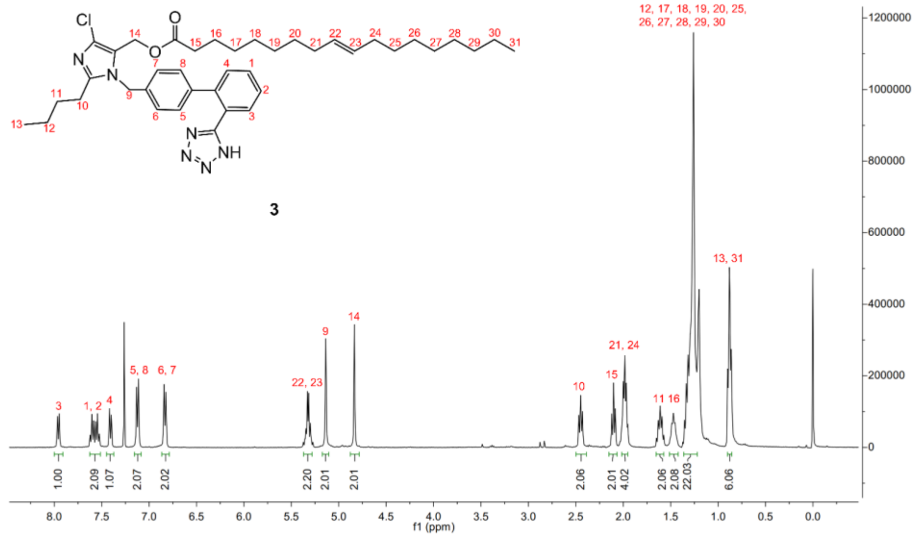
Cl
N
O
NH
3
12, 17, 18, 19, 20, 25,
26, 27, 28, 29, 30
13, 31
22, 23
9
14
5, 8
6, 7
3
1, 2
4
10
15
21, 24
11 16
f1 (ppm)
1200000
1000000
800000
600000
400000
200000
0
1.00 2.09 1.07 2.07 2.02 2.20 2.01 2.01 2.06 2.01 4.02 2.06 2.08 22.03 6.06

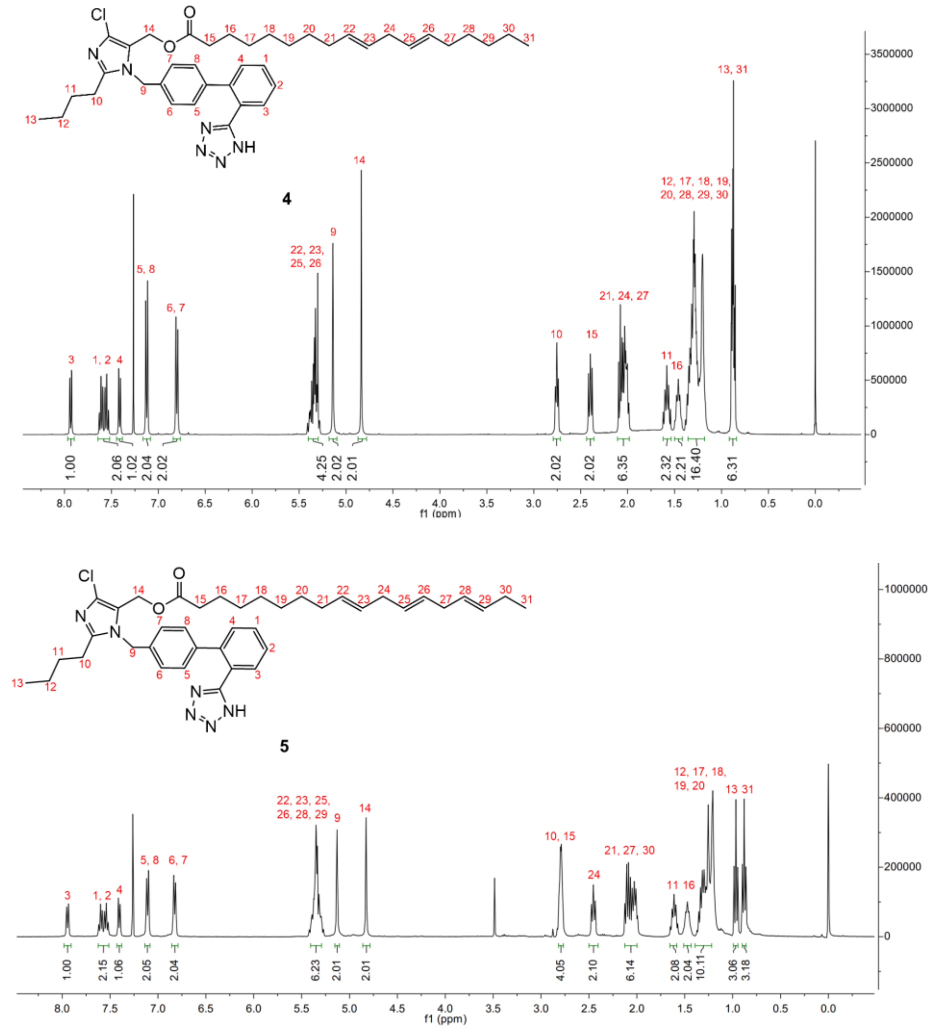

Figure S1. ^{1}H NMR spectrum of the losartan prodrugs 1-5 measured in $CDCl_3$. The numbers represent the protons in the conjugate.

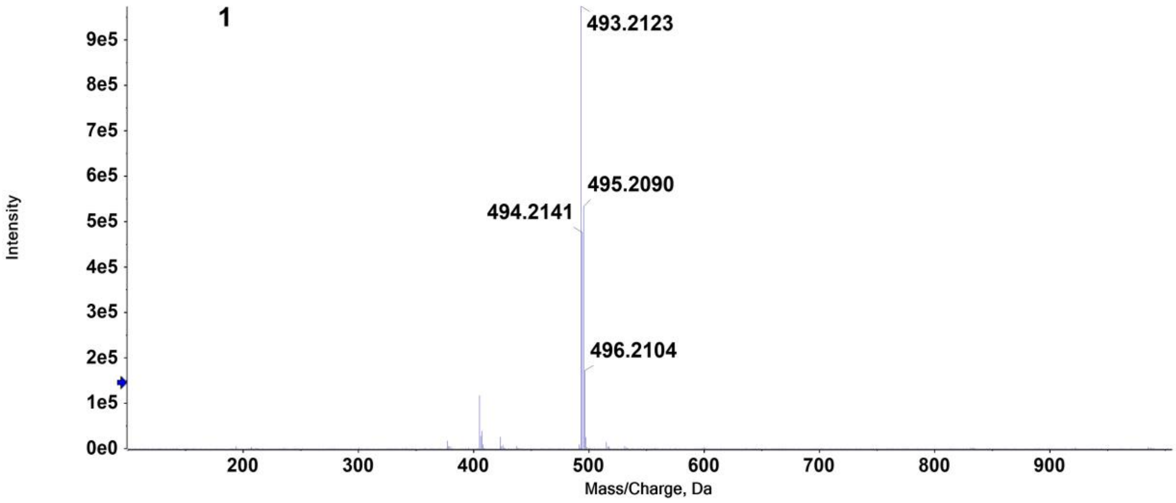
1
493.2123
495.2090
494.2141
496.2104
Intensity
9e5
8e5
7e5
6e5
5e5
4e5
3e5
2e5
1e5
0e0
200
300
400
500
600
700
800
900
Mass/Charge, Da

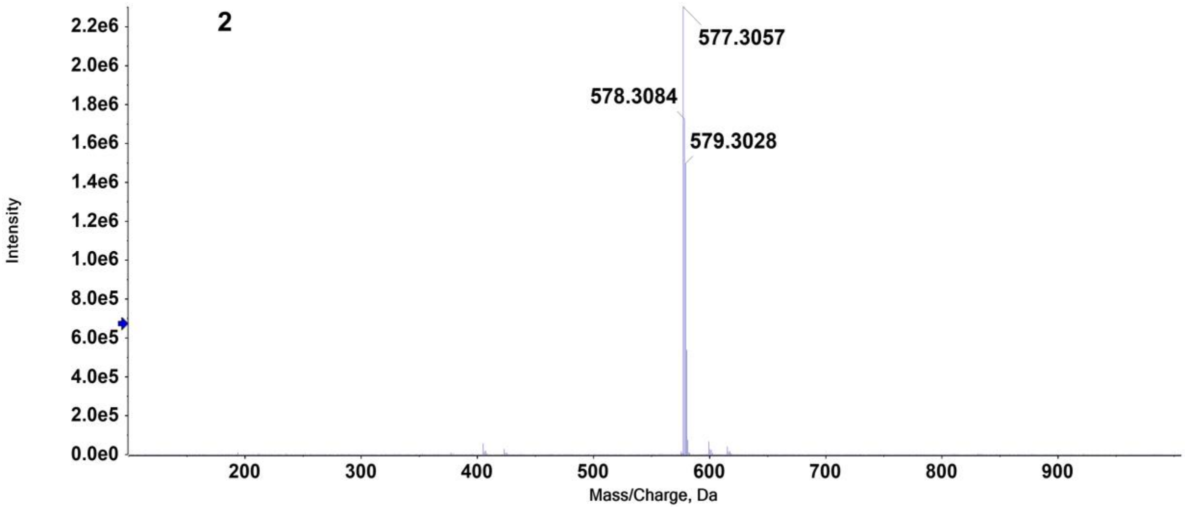
2
577.3057
578.3084
579.3028
Intensity
2.2e6
2.0e6
1.8e6
1.6e6
1.4e6
1.2e6
1.0e6
8.0e5
6.0e5
4.0e5
2.0e5
0.0e0
200
300
400
500
600
700
800
900
Mass/Charge, Da

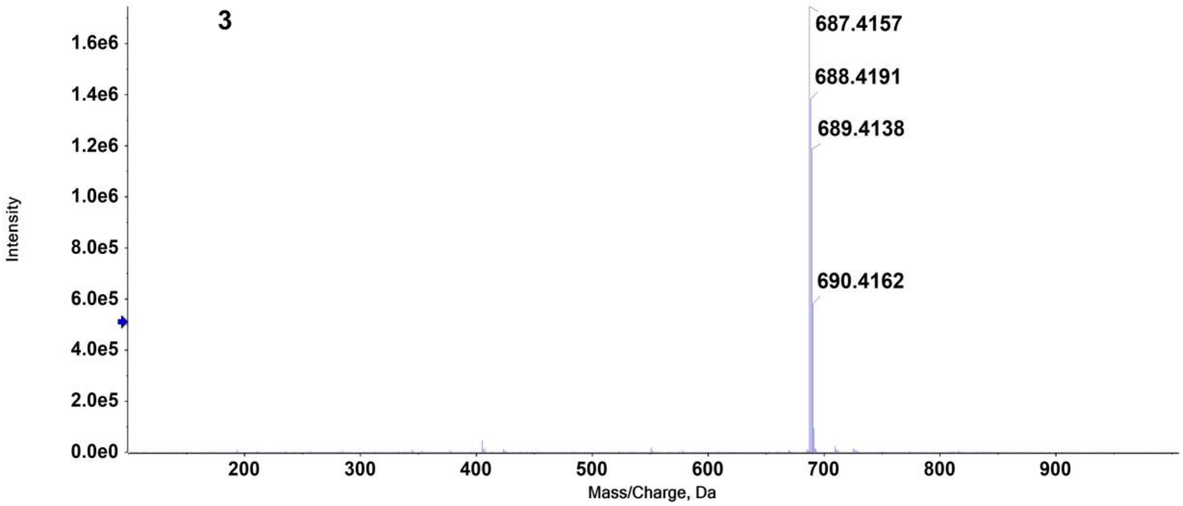
3
687.4157
688.4191
689.4138
690.4162
Intensity
1.6e6
1.4e6
1.2e6
1.0e6
8.0e5
6.0e5
4.0e5
2.0e5
0.0e0
200
300
400
500
600
700
800
900
Mass/Charge, Da

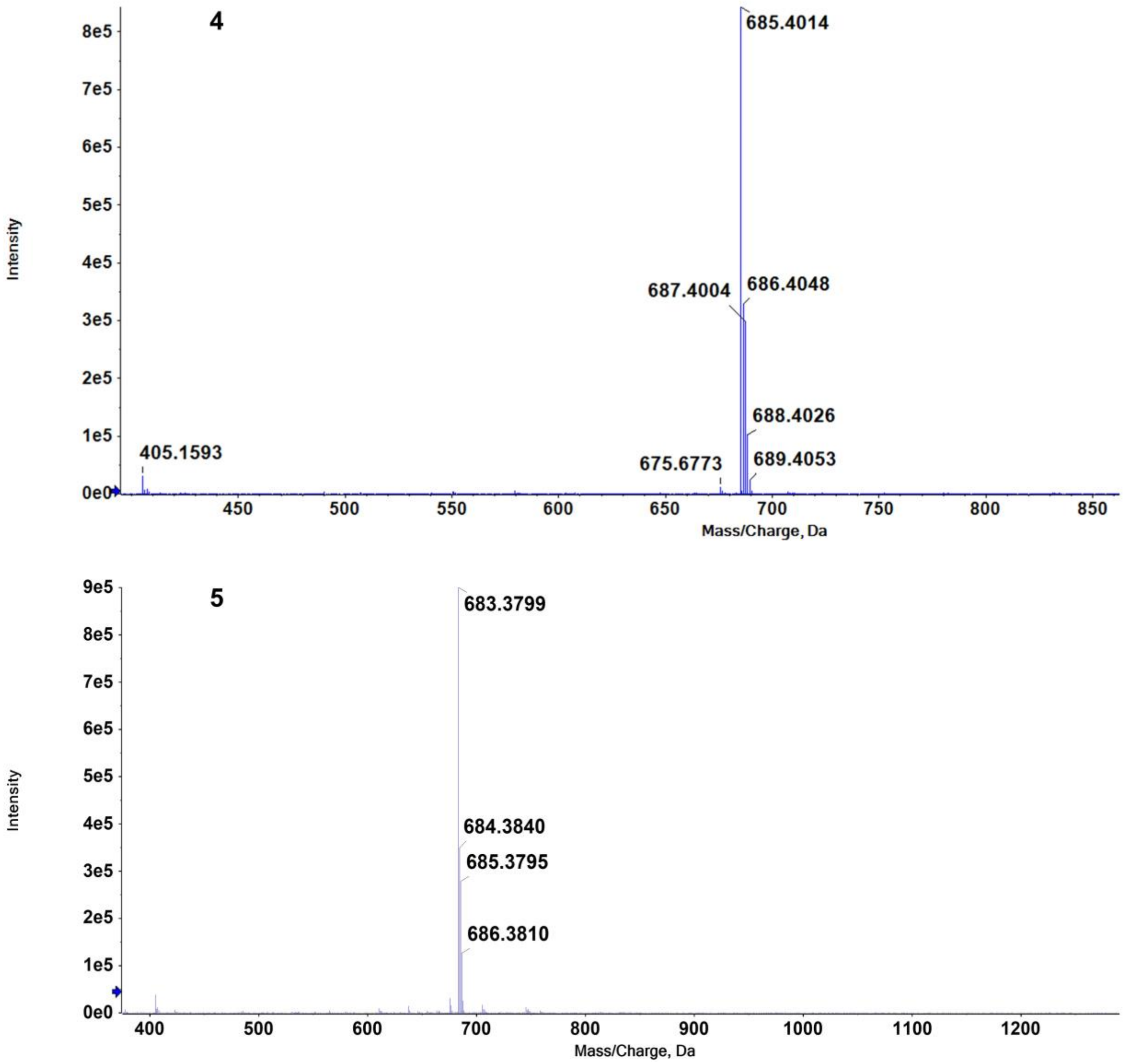

Figure S2. The high resolution mass spectrum of the losartan prodrugs 1-5.

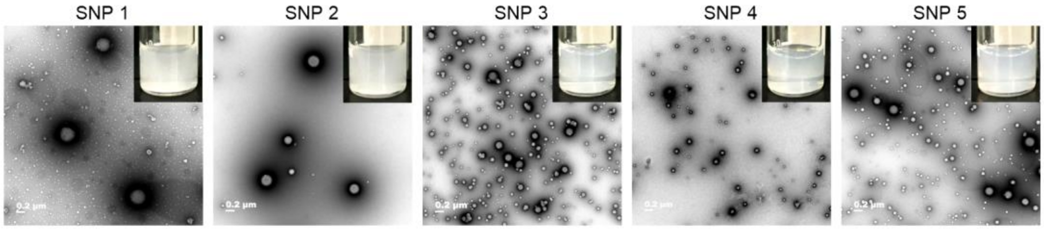

Figure S3. TEM images of nanoassemblies for prodrugs 1–5. The prodrugs **3**, **4** and **5** can form stable self-assembling nanostructures, but the prodrugs **1** and **2** are amenable to precipitate in water.

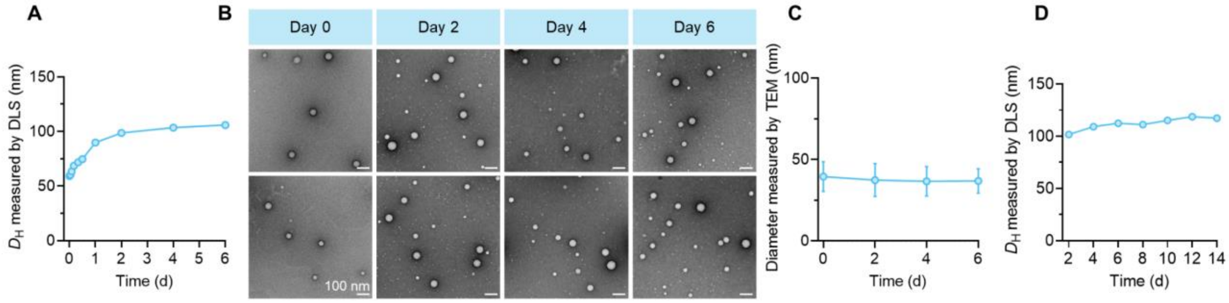

Figure S4. Dimensional stability of Los NB in water. A) Hydration diameter variation of Los NB in water from Day 0 to Day 6 by DLS analysis (n = 3). B) TEM images of Los NB in water at Day 0, 2, 4 and 6. C) Size of Los NB determined by TEM. D) Hydration diameter variation of Los NB in water from Day 2 to Day 14 by DLS analysis (n = 3). Data are presented as mean ± SD.

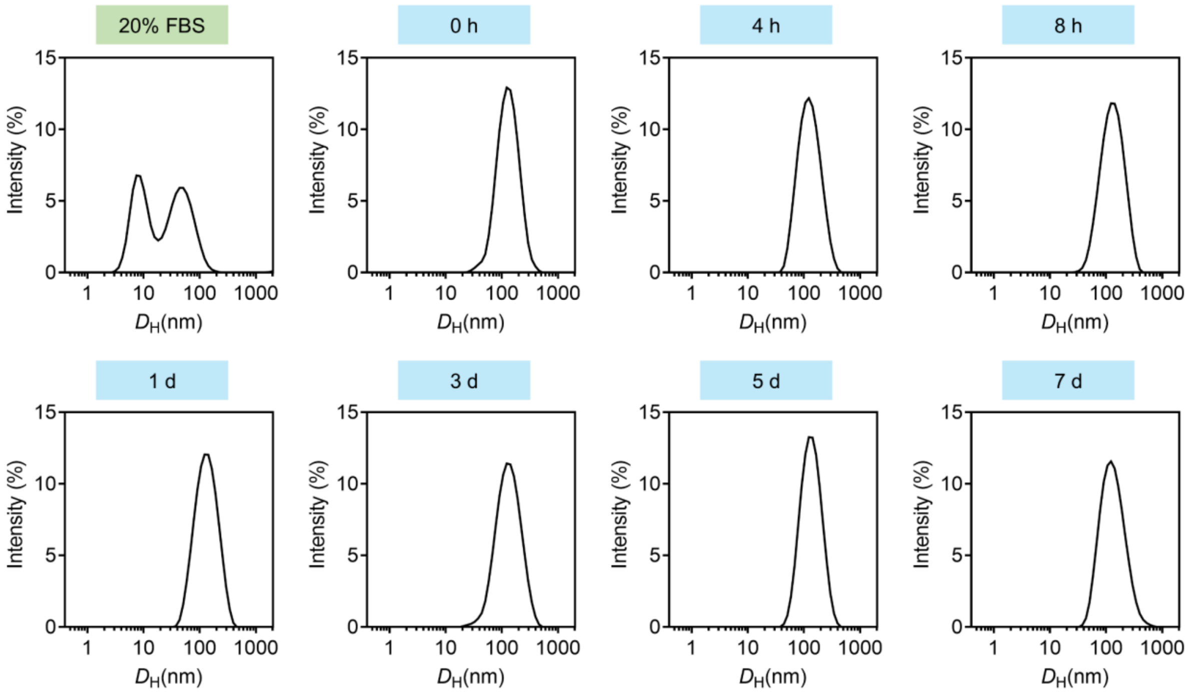

Figure S5. Diameter distributions of Los NB in 20% (v/v) FBS at different time points.

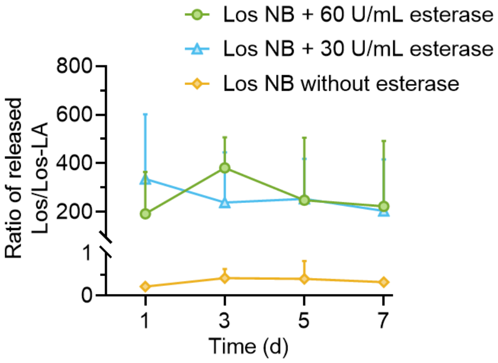

Figure S6. Ratio of released losartan/Los-LA from Los NB against PBS and PBS containing esterase (30 or 60 U/mL) (n = 3). Data are presented as mean ± SD.

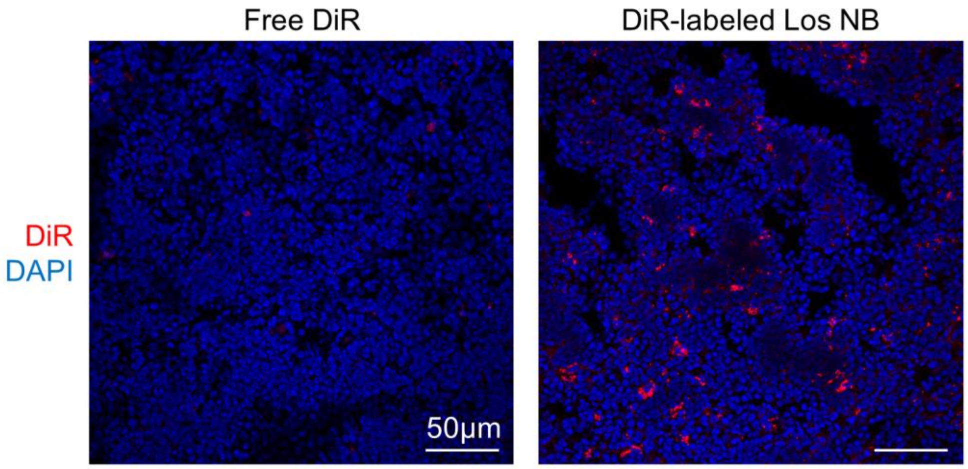

Figure S7. Penetration of free DiR and DiR-labeled Los NB in Panc02 tumor tissues at 24 h postadministration.

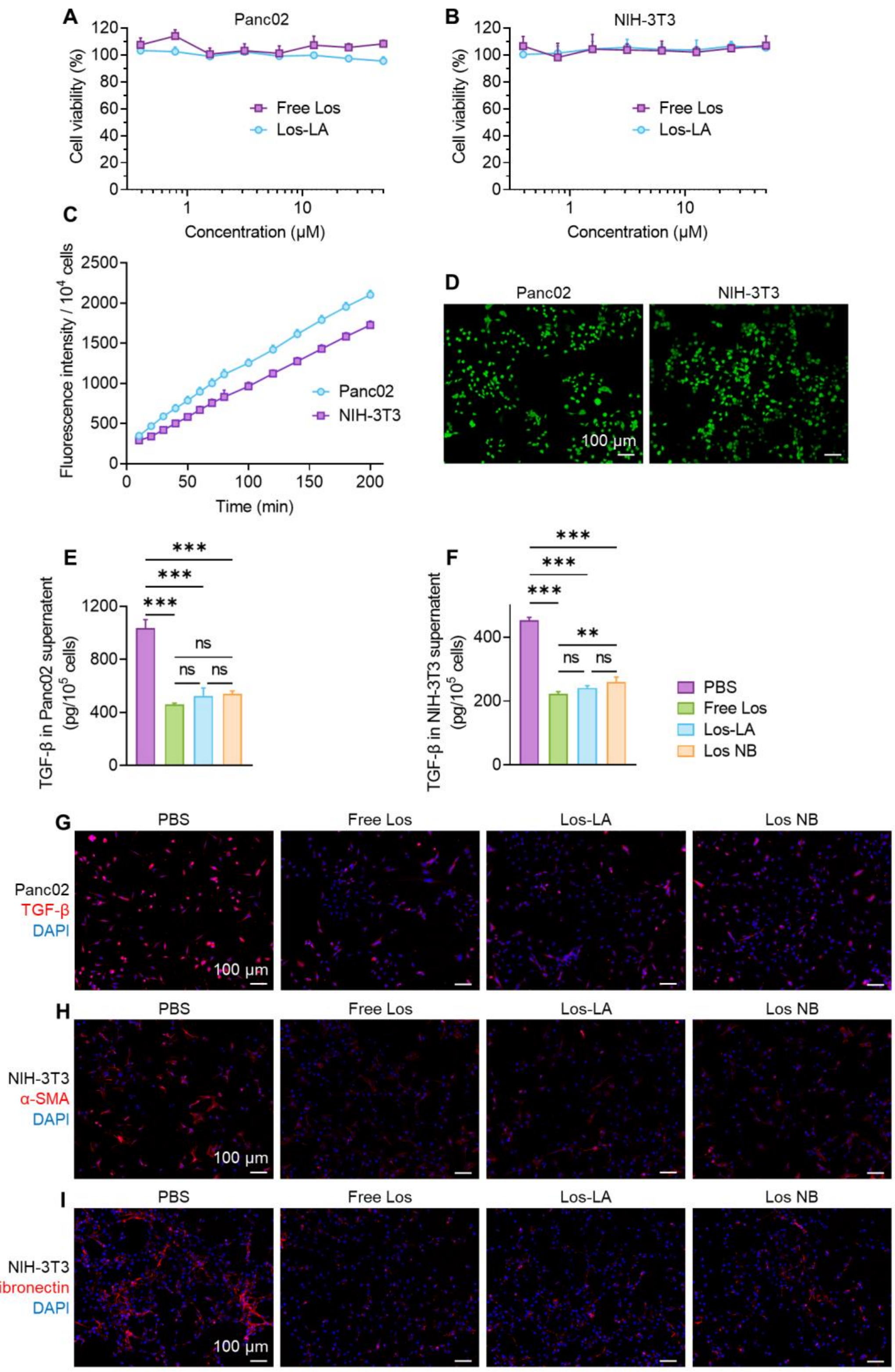

Figure S8. Stroma modulation activity of Los NB in vitro. A, B) Cytotoxicity of losartan and Los-LA in Panc02 tumor cells and NIH-3T3 fibroblasts after incubation for 24 h, and the cell

viabilities were determined by the CCK-8 assay (n = 4). C) Esterase activity of Panc02 and NIH-3T3 cells measured with fluorescein diacetate (FDA). Cells were plated into 96-well plates (1×10^4 cell per well). After 24 h, FDA was added to cell culture medium, and the FITC fluorescence intensity was detected at frequent intervals (Ex = 480 nm, Em = 530 nm) (n = 12). D) Representative fluorescent images of Panc02 and NIH-3T3 cells with FDA incubation for 30 min. E, F) The secretion of TGF-β in the supernatants of Panc02 and NIH-3T3 cells after different treatment evaluated by ELISA (n = 3). G-I) Immunofluorescent images of TGF-β in Panc02 cells, α-SMA and fibronectin in NIH-3T3 cells after different treatment. In E-I, cells were treated with PBS/free losartan/Los-LA/Los NB (20 μM losartan equivalent) for 24 h. Data are presented as mean ± SD. Significance was assessed by one-way ANOVA followed by LSD post hoc test. ns, $p > 0.05$, *$p < 0.05$, **$p < 0.01$, ***$p < 0.001$.

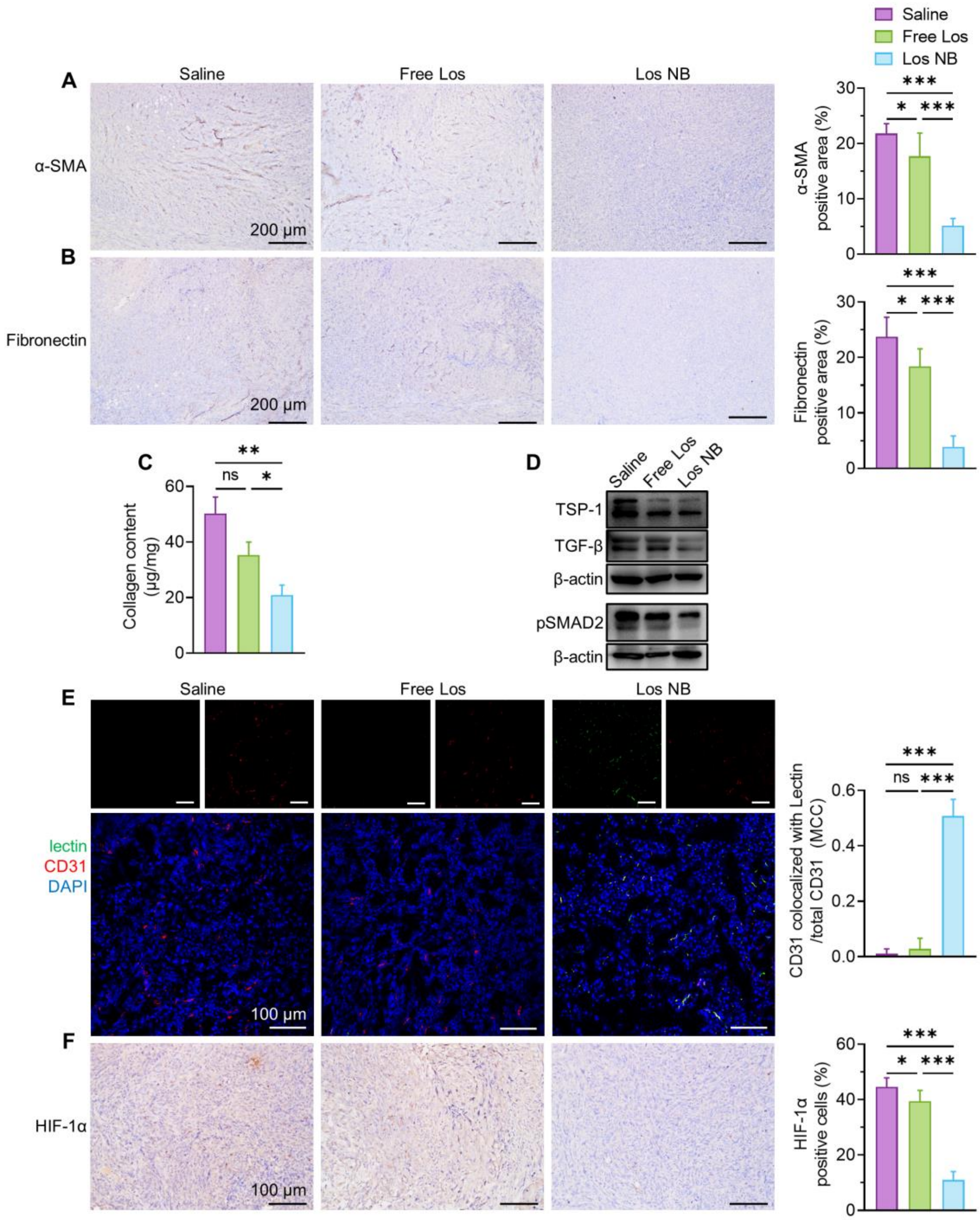

Figure S9. TME changes in Panc02 tumors after saline/free Los/Los NB treatments. A, B) Representative images and quantitative analysis of immunohistochemistry staining of α-SMA and fibronectin (n = 5). C) Collagen content in tumor tissues (n = 5). D) Western blot results of the expression of TSP-1, TGF-β and pSMAD2 of tumor tissues. E) Tumor vessel perfusion indicated by the percentage of perfused vessels (anti-CD31 staining colocalized with lectin) among total vessels (total CD31) (n = 5). (F) Representative images and quantitative analysis of hypoxia (anti-

HIF-1α staining) in tumor tissues (n = 5). Five randomly chosen visual fields were evaluated for histological quantification. Data are presented as mean ± SD. Significance was assessed by one-way ANOVA followed by LSD post hoc test. ns, $p > 0.05$, *$p < 0.05$, **$p < 0.01$, ***$p < 0.001$.

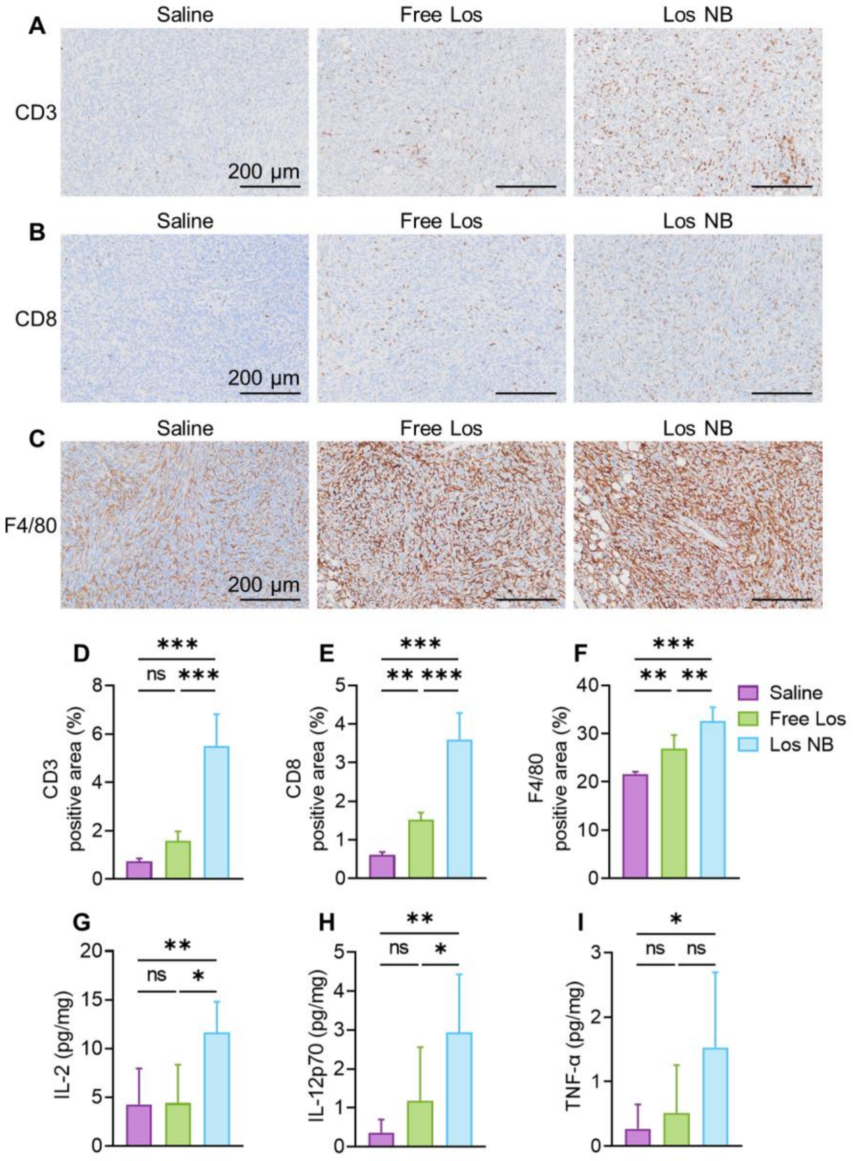

Figure S10. Immune microenvironment changes in Panc02 tumors after saline/free Los/Los NB treatments. A-F) Representative images and quantitative analysis of immunohistochemistry staining of $CD3^+$ T cells, $CD8^+$ T cells and $F4/80^+$ macrophages (n = 5). G-I) Content of IL-2, IL-12p70 and TNF-α in Panc02 tumor tissues (n = 5). Five randomly chosen visual fields were

evaluated for histological quantification. Data are presented as mean ± SD. Significance was assessed by one-way ANOVA followed by LSD post hoc test. ns, $p > 0.05$, *$p < 0.05$, **$p < 0.01$, ***$p < 0.001$.

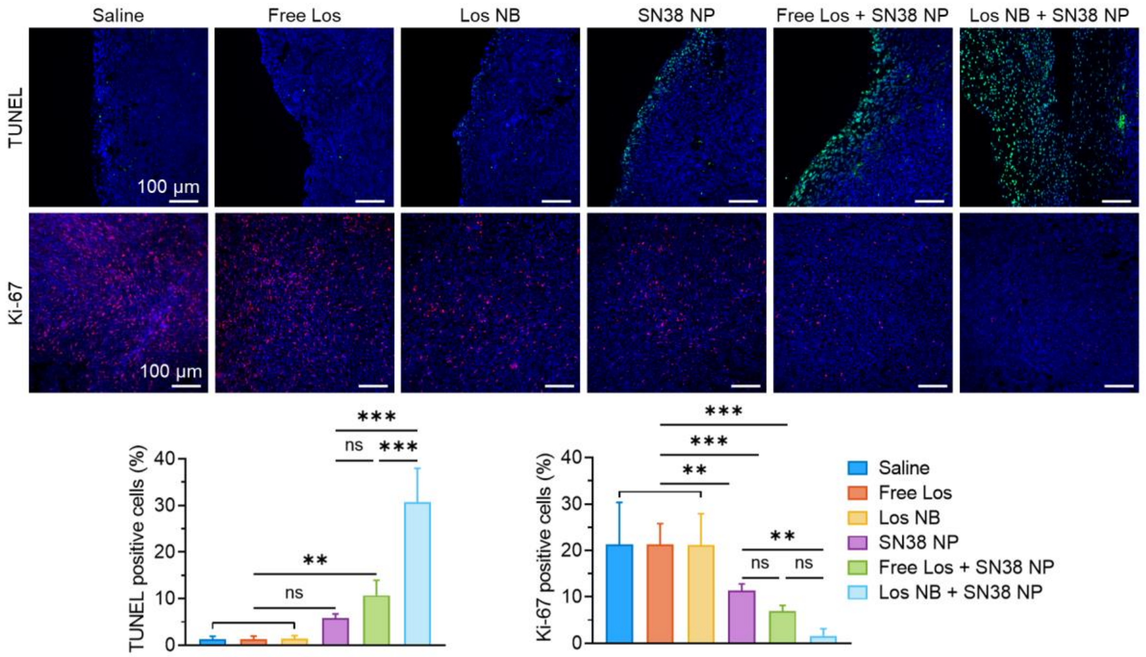

Figure S11. Representative TUNEL and Ki-67 staining images and quantitative analysis of Panc02 tumor tissues on Day 28 ($n = 5$). Five randomly chosen visual fields were evaluated for histological quantification. Data are presented as mean ± SD. Significance was assessed by one-way ANOVA followed by LSD post hoc test. ns, $p > 0.05$, *$p < 0.05$, **$p < 0.01$, ***$p < 0.001$.

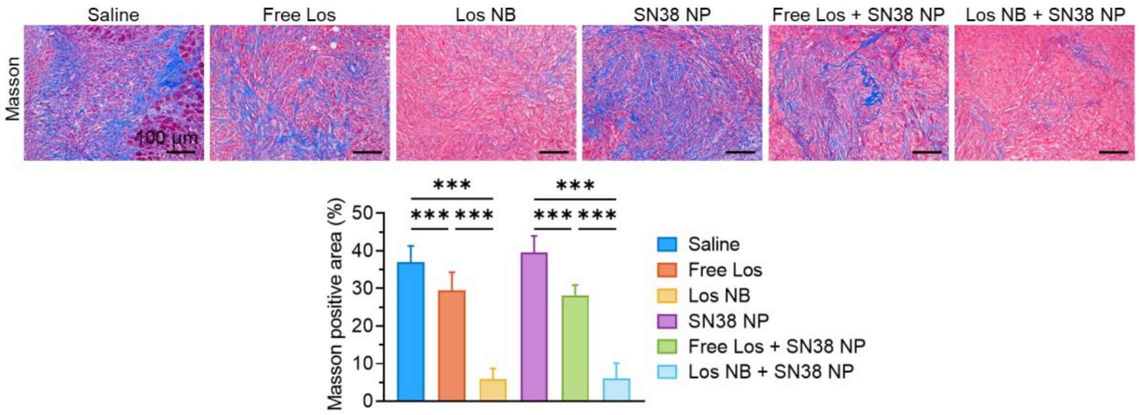

Figure S12. Representative Masson staining images and quantitative analysis of Panc02 tumor tissues on Day 28 (n = 5). Five randomly chosen visual fields were evaluated for histological quantification. Data are presented as mean ± SD. Significance was assessed by one-way ANOVA followed by LSD post hoc test. ns, $p > 0.05$, *$p < 0.05$, **$p < 0.01$, ***$p < 0.001$.

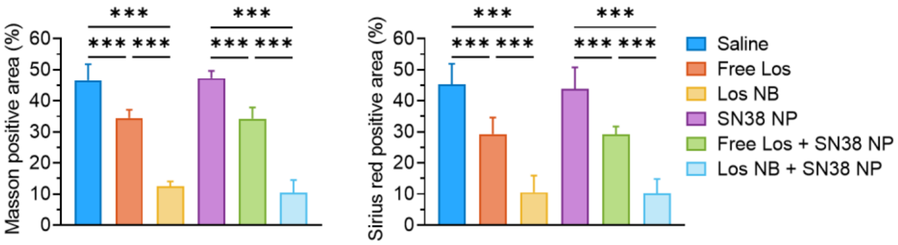

Figure S13. Quantification of Masson and Sirius red staining of PDX tumor tissues on Day 28 (n = 5). Five randomly chosen visual fields were evaluated for histological quantification. Data are presented as mean ± SD. Significance was assessed by one-way ANOVA followed by LSD post hoc test. ns, $p > 0.05$, *$p < 0.05$, **$p < 0.01$, ***$p < 0.001$.

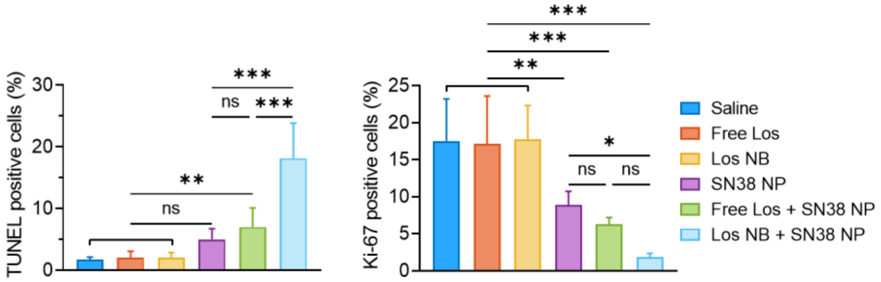

Figure S14. Quantification of TUNEL and Ki-67 staining of PDX tumor tissues on Day 28 (n = 5). Five randomly chosen visual fields were evaluated for histological quantification. Data are presented as mean ± SD. Significance was assessed by one-way ANOVA followed by LSD post hoc test. ns, $p > 0.05$, *$p < 0.05$, **$p < 0.01$, ***$p < 0.001$.

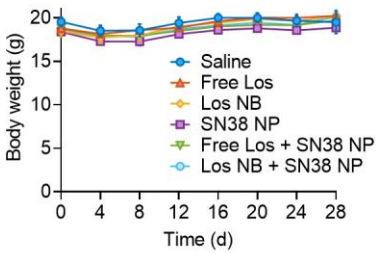

Figure S15. Body weight changes in Panc02 model mice (n = 5). Data are presented as mean ± SD.

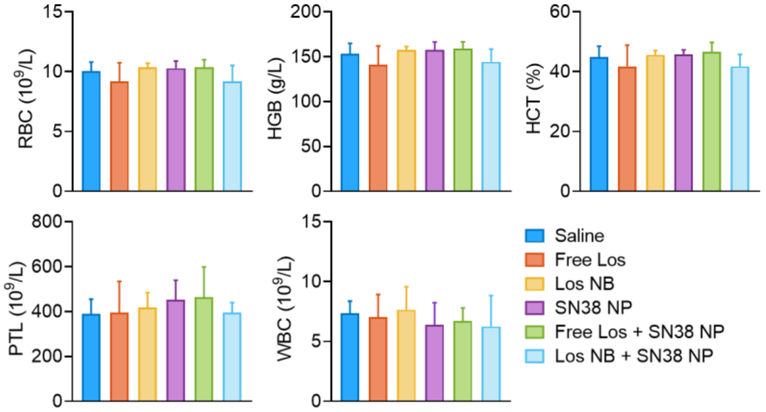

Figure S16. The analysis of blood routine, including RBC, HGB, HCT, WBC and PLT of the

Panc02 model mice on Day 28 (n = 5). Data are presented as mean ± SD.

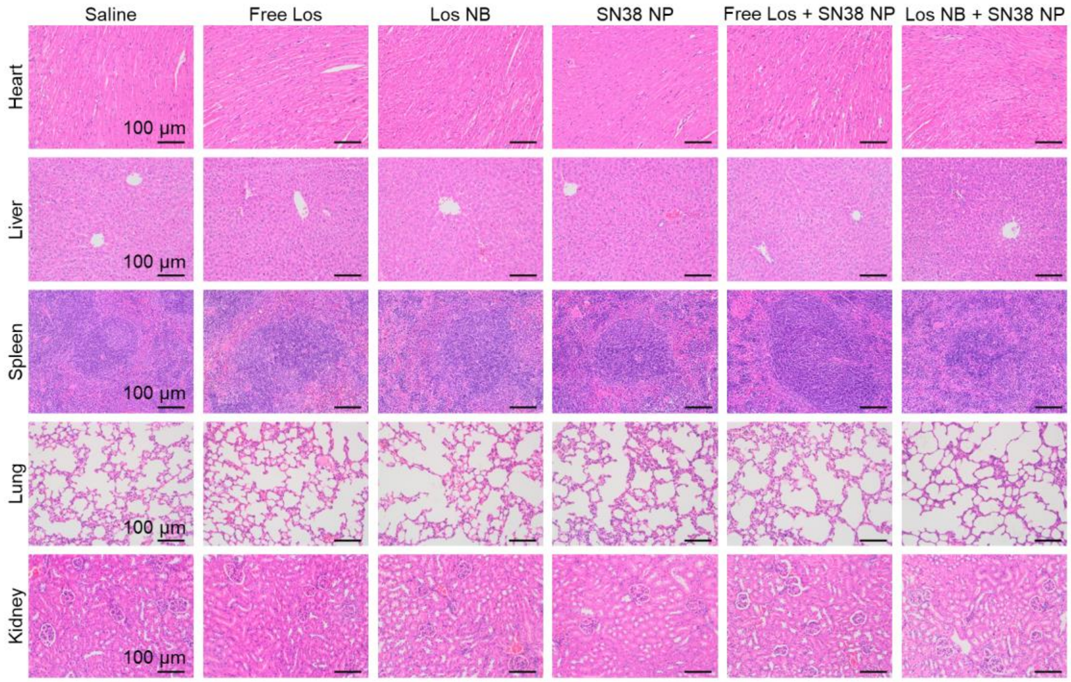

Figure S17. Representative H&E staining images of heart, liver, spleen, lung and kidney of the Panc02 model mice on Day 28.

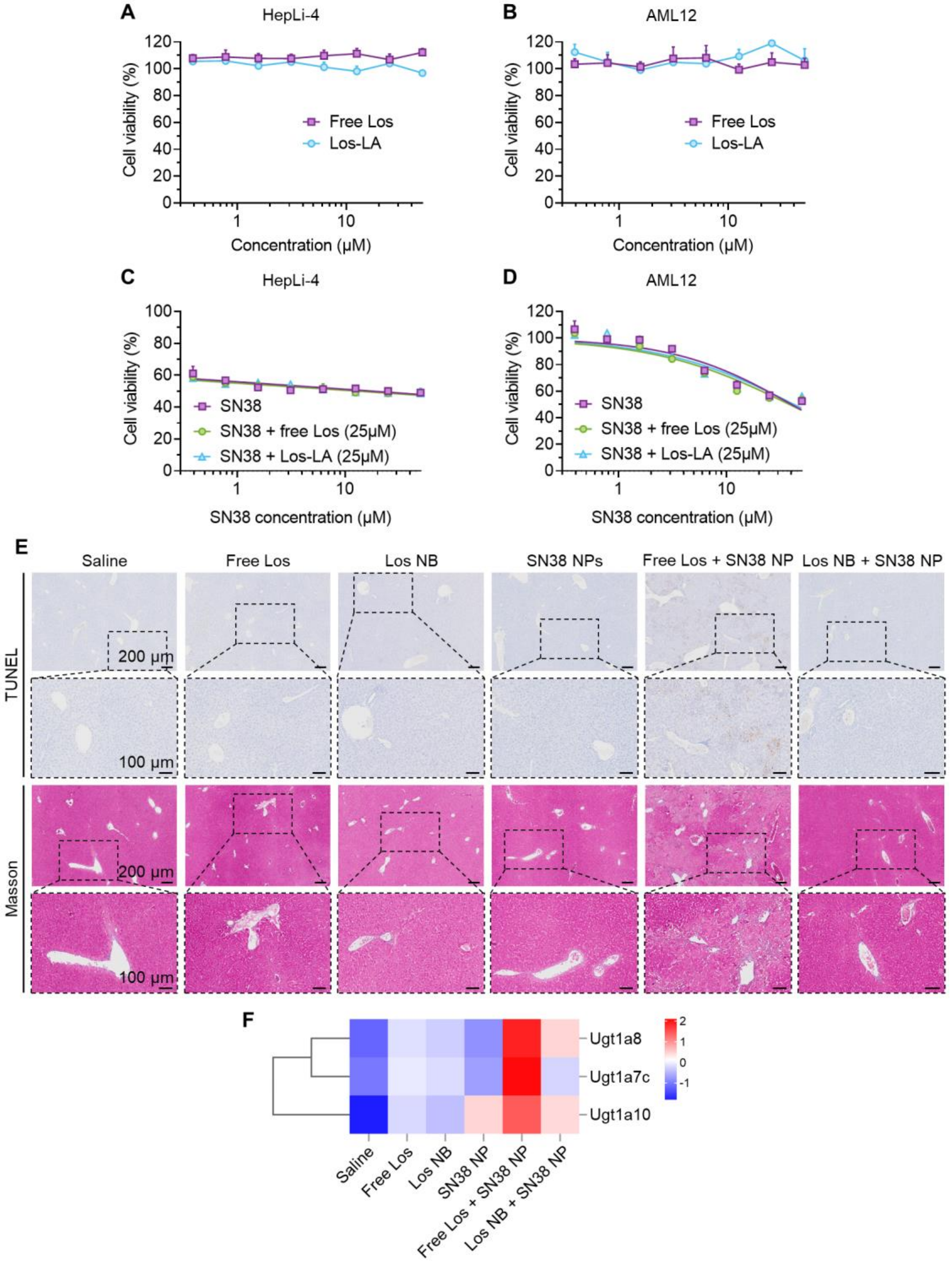

Figure S18. Exploration of reasons behind the hepatotoxicity caused by free Los with SN38 NP treatment. A, B) In vitro cytotoxicity of losartan and Los-LA in human HepLi-4 hepatocytes and mouse AML12 hepatocytes after incubation for 24 h (*n* = 4). C, D) In vitro synergistic cytotoxicity of SN38 combined with losartan or Los-LA in HepLi-4 and AML12 hepatocytes (*n* =

4). In A-D, the cell viabilities were determined by the CCK-8 assay. E) liver sections staining images of hepatocyte apoptosis (TUNEL) and fibrosis (Masson) in mice undergoing complete treatment. F) The heat map for the gene expression level of UDP-glucuronosyltransferase A1 (UGT1A) in livers by RNA sequencing (n = 3). Data are presented as mean ± SD.

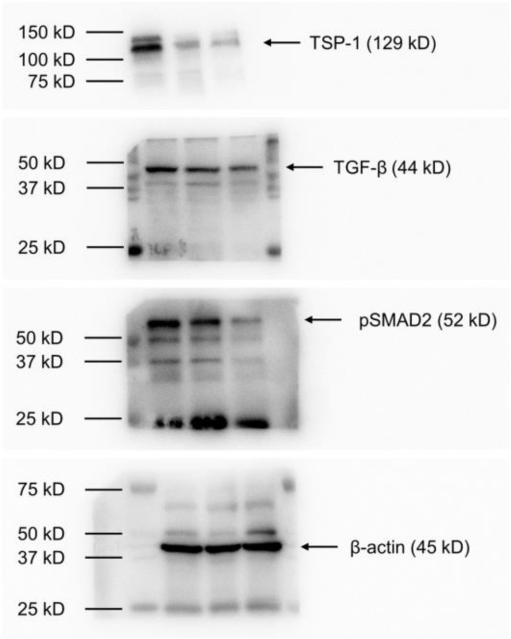

Figure S19. Original photographs of western blotting data in Figure 3G.

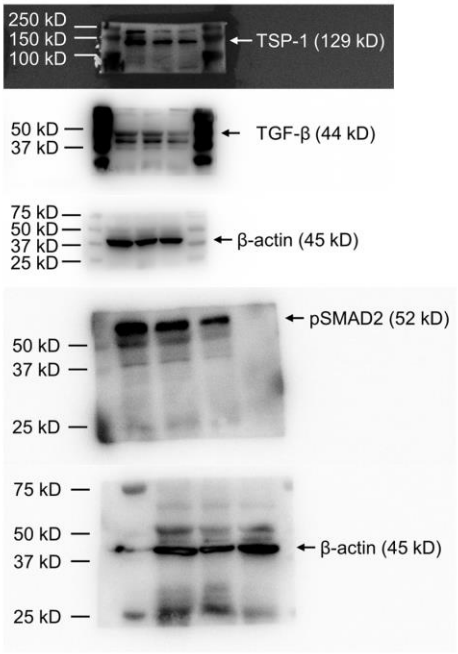

Figure S20. Original photographs of western blotting data in Figure S9D, Supporting Information.

Table S1. Antibodies used in this study.

Antibodies	Company	Catalog	Application
Anti-α-SMA	HuaBio	ET1607-53	IF/IHC
AF594 Anti-CD31	Biolegend	102432	IF
Anti-Ki-67	ABclonal	A20018	IF
Anti-fibronectin	Abcam	ab268020	IHC
Anti-HIF-1α	Abcam	ab113642	IHC
Anti-CD3	Abcam	ab237721	IHC
Anti-CD8	Abcam	ab209775	IHC
Anti-F4/80	CST	70076	IHC
Anti-TSP-1	Abcam	ab267388	WB
Anti-TGF-β	ABclonal	A15103	WB
Anti-pSMAD2	Abcam	ab280888	WB
Anti-β-actin	CST	3700	WB
Goat anti-rabbit HRP	CST	7074	WB
Goat anti-mouse HRP	CST	7076	WB
